# The carboxyl-terminal TSP1-homology domain is the biologically active effector peptide of matricellular protein CCN5 that counteracts profibrotic CCN2

**DOI:** 10.1016/j.jbc.2022.102803

**Published:** 2022-12-15

**Authors:** Sima Zolfaghari, Ole Jørgen Kaasbøll, Vivi T. Monsen, Bojana Sredic, Else Marie V. Hagelin, Håvard Attramadal

**Affiliations:** 1 Institute for Surgical Research, Oslo University Hospital, Oslo, Norway; 2 Institute of Clinical Medicine, University of Oslo, Oslo, Norway

**Keywords:** CCN5, WISP2, TSP1-homology domain, CCN3, CCN2, matricellular protein, fibroblasts, primary human lung fibroblasts, cell migration, phosphokinase signaling, phospho-ERK1/2, AKT, AKT serine/threonine protein kinase/protein kinase B, ATCC, American Type Culture Collection, CCN, Cellular Communication Network, CK, cystine knot, DMEM, Dulbecco’s modified Eagle’s medium, EMT, epithelial-to-mesenchymal transition, ERK, extracellular signal-regulated kinase, ER-α, estrogen-receptor α, FBS, fetal bovine serum, Hevin, high endothelial venule protein, HHS, His-Halo-Sumo, HTRF, homogenous time-resolved fluorescence, ID2, inhibitor of DNA binding 2, MEK, mitogen-activated protein kinase kinase, MMP, matrix metalloproteinase, SPARC, secreted protein, acidic, and rich in cysteine, Sumo, small ubiquitin-like modifier protein (from *Saccharomyces cerevisiae*), TGF, transforming growth factor, TSP1, thrombospondin type 1 repeat, VEGF, vascular endothelial growth factor, vWC, von Willebrand factor type C repeat, WISP2, WNT1-inducible signaling pathway protein 2

## Abstract

Cellular Communication Network (CCN) proteins have multimodular structures important for their roles in cellular responses associated with organ development and tissue homeostasis. CCN2 has previously been reported to be secreted as a preproprotein that requires proteolytic activation to release its bioactive carboxyl-terminal fragment. Here, our goal was to resolve whether CCN5, a divergent member of the CCN family with converse functions relative to CCN2, releases the TSP1 homology domain as its bioactive signaling entity. The recombinant CCN5 or CCN3 TSP1 homology domains were produced in ExpiCHO-S or DG44 CHO cells as secretory fusion proteins appended to the carboxyl-terminal end of His-Halo-Sumo or amino-terminal end of human albumin and purified from the cell culture medium. We tested these fusion proteins in various phosphokinase signaling pathways or cell physiologic assays. Fusion proteins with the CCN5 TSP1 domain inhibited key signaling pathways previously reported to be stimulated by CCN2, irrespective of fusion partner. The fusion proteins also efficiently inhibited CCN1/2-stimulated cell migration and gap closure following scratch wound of fibroblasts. Fusion protein with the CCN3 TSP1 domain inhibited these functions with similar efficacy and potency as that of the CCN5 TSP1 domain. The CCN5 TSP1 domain also recapitulated a positive regulatory function previously assigned to full-length CCN5, that is, induction of estrogen receptor-α mRNA expression in triple negative MDA-MB-231 mammary adenocarcinoma cells and inhibited epithelial-to-mesenchymal transition and CCN2-induced mammosphere formation of MCF-7 adenocarcinoma cells. In conclusion, the CCN5 TSP1 domain is the bioactive entity that confers the biologic functions of unprocessed CCN5.

Cellular Communication Network factors (CCN proteins) are a family of six highly conserved matricellular proteins involved in intercellular and cell–matrix communication (1, 2). CCN proteins are secreted proteins thought to play important roles in the homeostasis of parenchymal tissues. Notably, CCN proteins are most active during organ development and following tissue injury (2). Structurally, CCN proteins are multimodular proteins consisting of four distinct structural domains (except for CCN5 which lacks the carboxyl-terminal fourth domain). From the amino-terminal end, the four domains are an insulin-like growth factor–binding protein homology domain, a von Willebrand factor type C repeat (vWC) homology domain, a thrombospondin type 1 (TSP1) repeat homology domain, and a carboxyl-terminal domain with a cystine knot motif (CK) (3, 4, 5). The two latter domains are separated from the two former amino-terminal domains by an unstructured “hinge region”, reported to be sensitive to various endopeptidases, including several members of the matrix metalloproteinase family (MMPs) (6). The crystal structure of the vWC and TSP1 domains of CCN3 have been reported, providing some structural insights with implications for all CCN family proteins (7, 8). However, the overall globular structure is still missing. According to the prevailing view, the multimodular structure of the CCN proteins allows them to interact with various growth factors and other proteins in the extracellular matrix to control and orchestrate complex cellular responses (3, 9). Nonetheless, it is well known that CCN proteins may also engender rapid signaling responses and are likely autocrine/paracrine factors in their own right, activating plasma membrane receptors that transmit signals into the cell. Yet, direct receptors for these proteins that conform to pharmacologic criteria have so far not been identified. Missing identification of cognate receptors for CCN proteins conceivably could be due to the fact that knowledge on the bioactive signaling entity of CCN proteins for long has remained unknown. To this end, we recently reported that CCN2 is secreted as a preproprotein that needs to undergo endopeptidase cleavage in order to release the biologically active fragment, that is, the carboxyl-terminal fragment consisting of the TSP1 and CK domains (10). Furthermore, we showed that this carboxyl-terminal fragment of CCN2 elicits rapid signaling responses and confers a broad spectrum of biological actions previously assigned to full-length CCN2. We also provided evidence that similar carboxyl-terminal fragments of CCN1 and CCN3 are fully active biological entities (10). However, it is still unknown to what extent CCN5 (also called WISP2), a divergent member of the CCN family lacking the carboxyl-terminal CK domain may undergo proteolytic activation. Several MMP isoforms have been shown to cleave CCN5 in the hinge region (6). However, to what extent and in what ways such cleavage may alter the biological activities of CCN5 remain to be resolved. CCN5 is particularly interesting in the sense that it has been reported to confer converse or opposite actions to those of the 4-domain CCN proteins (11, 12). For example, CCN5 has been shown to inhibit the profibrotic actions of CCN2 (13). Thus, the aims of this study was to resolve to what extent the carboxyl-terminal TSP1 domain of CCN5 is a biologically active entity sufficient in conveying the signaling and biological actions of CCN5. Since CCN2 has previously been documented to be essential to the function of fibroblasts (14), we focused on the ability of the TSP1 domain of CCN5 to inhibit CCN2-stimulated migration and contraction of fibroblasts. Furthermore, as the MEK/ERK1/2 pathway has been shown to be a key signaling pathway involved in collective directional cell migration, as typically takes place in closure of *in vitro* scratch wounds, we also interrogated CCN5-mediated inhibition of ERK1/2 activities in this assay. Although the mechanisms are not quite clear, CCN3 or fragments of CCN3 have likewise been reported to counteract CCN2 and to inhibit fibroblast functions and fibrosis (15, 16). As the TSP1 domain of CCN3 is highly similar to that of CCN5, an important issue to address was to what extent the TSP1 domains of CCN3 and CCN5 would display functional similarities. Thus, we investigated in parallel the signaling and biological actions of these two domains. Following the recent report of the crystal structure of the TSP1 domain of CCN3 (8), functional differences among these domains might then be related to specific structural dissimilarities. The recombinant TSP1 domains were expressed as fusion proteins with various fusion partners in order to improve expression, secretion, solubility, and stability. These fusion partners have also been shown to facilitate efficient purification of the proteins (17, 18). The recombinant proteins were produced for secretion from stably transfected CHO cells adapted for suspension culture and purified from the cell culture medium by sequential fast protein liquid chromatography including affinity chromatography and size-exclusion chromatography and subsequently assessed for cell signaling activity and cell biologic functions in experiments *in vitro*.

## Results

### Production and purification of recombinant fusion proteins with the TSP1 domain of CCN5 or CCN3

Production of separate recombinant TSP1 homology domains of CCN5 or CCN3 engineered for secretion from eukaryotic expression systems proved to be difficult due to very poor yield, challenges regarding solubility, and uncertainty regarding correct protein folding. Thus, in order to produce the recombinant TSP1 domain of CCN5 and CCN3 appropriate and sufficient for studies of its biologic activities and functions, we decided to make fusion proteins with the TSP1 domain of CCN5/3 appended either to a complex consisting of 6 × histidines, Halo-tag and Sumo (His-Halo-Sumo (HHS)), or to human albumin. Halo-tag is a 33 kDa peptide fragment derived from prokaryotic haloalkane dehydrogenase that may form covalent bonds with chloroalkane-linked tags enabling efficient purification. The Sumo protein is a small ubiquitin-like modifier protein from *Saccharomyces cerevisiae* that when used as an N-terminal fusion partner functions both as a chaperonin and initiator of protein folding both in prokaryotic and eukaryotic systems (19, 20). In this context, Sumo has also been shown to significantly improve protein stability and solubility of recombinant proteins (17). Initially, our plan was to remove the HHS tag following purification of TSP1 fusion protein by incubation with Sumo-specific protease. However, the latter reaction was not quantitative. Thus, we decided to leave the HHS tag in place and in addition, make recombinant TSP1 of CCN5/3 aminoterminally fused to human albumin in order to have two different fusion partners serve as internal controls. Full-length CCN5 was also generated as fusion protein with HHS (HHS-CCN5(FL)) to serve as control for HHS-CCN5(TSP1) (HHS-CCN5(III)). In order to control for possible effects conferred by the fusion partner, recombinant HHS fusion partner with signal peptide for secretion was also expressed in CHO cells similar to the fusion proteins with CCN(TSP1) domains and purified from the cell culture medium. Plasmid maps of the expression vectors used for generation of stably transfected CHO cells (DG44 CHO or ExpiCHO) expressing the various fusion proteins or fusion partner are shown in Fig. S1, *A* and *B*. Schematic diagrams of the chromatographic purification of the various fusion proteins and fusion partner are provided in Fig. S1*C* with details regarding chromatographic columns, and binding and elution buffers in Table S1. In order to assess purity, the purified recombinant proteins were subjected to SDS-PAGE using TGX Stain-Free Protein Gels (Bio-Rad) as shown in Fig. S1, *D* and *E*. The final polishing step by size-exclusion chromatography was necessary to remove coeluting TGF-β (transforming growth factor-β), which frequently has been found to copurify with recombinant proteins from CHO cells (10, 21, 22). Residual TGF-β activity in the final recombinant protein preparations was assessed by ELISA of TGF-β and revealed extremely low levels not sufficient to induce SMAD-reporter activities (10).

### The TSP1 domain is the bioactive signaling entity of CCN5

**Figure 1:**
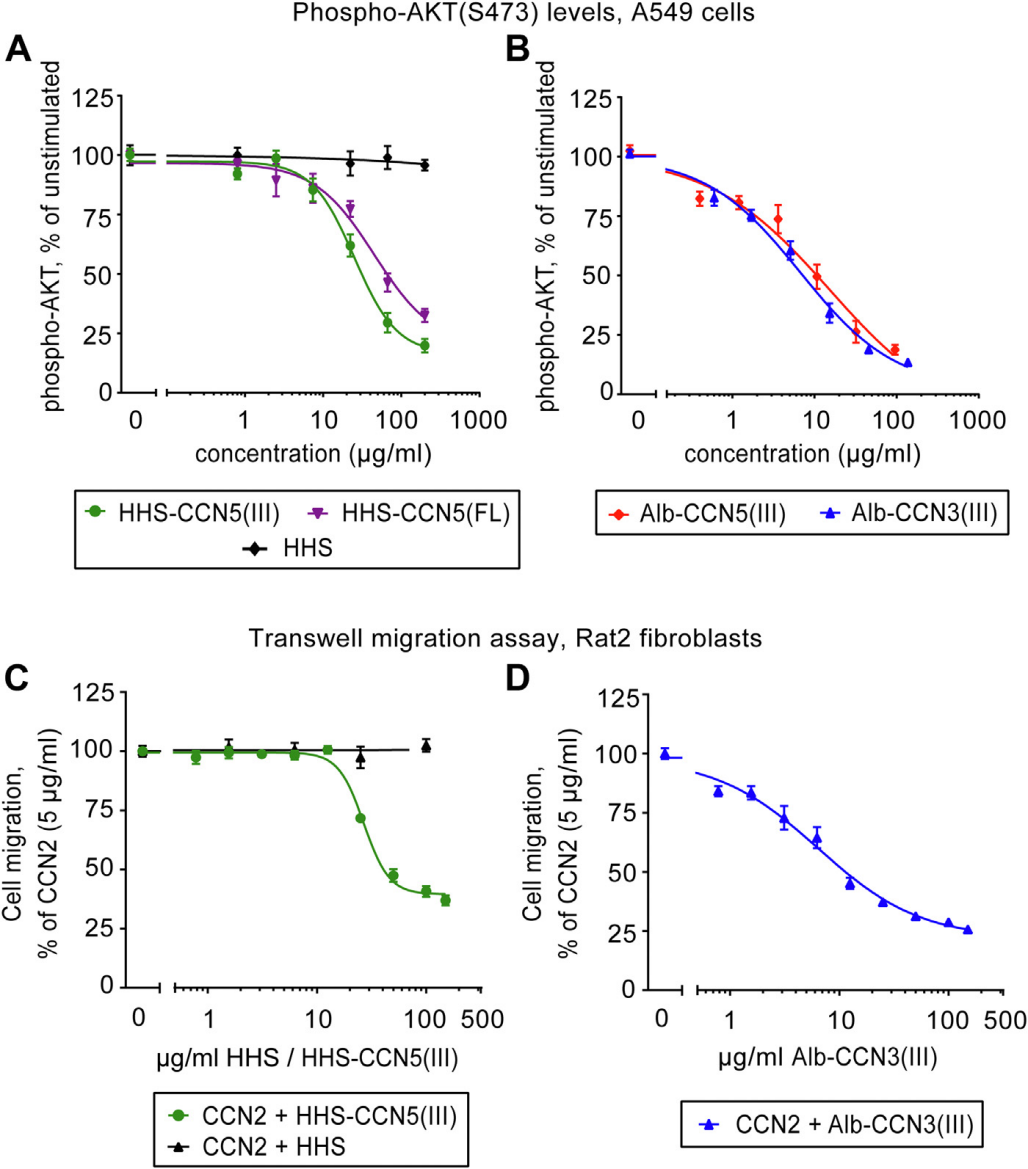
**The TSP1 domain of CCN5 is sufficient to inhibit phosphorylation of AKT and CCN2-induced cell migration.** Panel (*A*) demonstrates concentration-effect curves of HHS-CCN5(III)- and HHS-CCN5(FL)-mediated inhibition of phospho-AKT (Ser473) levels in A549 human adenocarcinoma epithelial cells following 60 min incubation with the indicated proteins. The His-Halo-Sumo (HHS) fusion partner alone did not display any efficacy. Panel (*B*) demonstrates concentration-effect curves of Alb-CCN5(III)- and Alb-CCN3(III)-stimulated inhibition of phospho-AKT (Ser473) levels in A549 cells following 60 min incubation with the indicated proteins. The data in panels (*A* and *B*) represent the mean ± SEM (n = 3 independent experiments assayed in three replicates for each data point) using a TR-FRET–based or AlphaLISA-based immunoassay of cellular phospho-AKT (Ser473) contents. The data were subjected to curve fitting by four-variable nonlinear regression in GraphPad Prism. Panels (*C*) and (*D*) demonstrate concentration-effect curves of HHS-CCN5(III)- and Alb-CCN3(III)-mediated inhibition of CCN2 (5 μg/ml)-stimulated migration of Rat2 fibroblasts following 20 h incubation. The HHS fusion partner alone did not show any efficacy in the migration of Rat2 fibroblasts. Cell migration was determined using multiscreen migration assay (Boyden chamber principle). The data represent the mean ± SEM (n = 3 independent experiments assayed in four replicates for each data point). The data were subjected to curve fitting by four-variable nonlinear regression in GraphPad Prism. Alb, albumin; CCN, Cellular Communication Network; HHS, His-Halo-Sumo; TSP1, thrombospondin type 1 repeat.

Current knowledge on intracellular signaling cascades activated by CCN5 is fragmentary. However, it is well documented that CCN5 may inhibit phosphorylation of AKT in both estrogen-receptor α (ER-α)–negative and ER-α–positive breast cancer cells as well as in certain other cancer cell lines (23, 24, 25, 26). In order to resolve to what extent full-length CCN5 may be considered a latent pro-form and the carboxyl-terminal TSP1-domain being the fully active entity that confers the range of biologic activities previously reported for full length CCN5, we assessed intracellular phosphokinase activities (phospho-AKT levels) and various cell biologic responses following exposure of cells to full-length CCN5 or the TSP1 domain of CCN5. First, we investigated to what extent full-length CCN5 (HHS-CCN5(FL)) or the TSP1 domain of CCN5 (HHS-CCN5(III)) would inhibit phospho-AKT (Ser473) levels in A549 human lung adenocarcinoma cells. This cell line was chosen because it displays high basal phospho-AKT (Ser473) levels not responsive to further exogenous exposure to CCN2 (Fig. S2*A*) and CCN2 mRNA levels twice that of CCN5 mRNA according to The Human Protein Atlas databank registry (https://www.proteinatlas.org). Thus, we hypothesized that this cell line would be ideally suited for studies of CCN5(FL)- or CCN5(III)-mediated inhibition of phospho-AKT (Ser473) levels. As shown in Fig. S2*B*, we confirm that A549 cells are amenable to robust inhibition of phospho-AKT (Ser473) levels in the presence of the TSP1 domain of CCN5 (HHS-CCN5(III)). Basal phospho-AKT (Ser473) levels were also investigated in two other cell lines used in this study, that is, Rat2 fibroblasts and MCF-7 human mammary adenocarcinoma cells. Rat2 fibroblasts display extremely low basal phospho-AKT (Ser473) levels and high sensitivity to exogenous exposure to CCN2, whereas MCF-7 cells with CCN5 mRNA levels two times higher than that of CCN2 mRNA (Human Protein Atlas databank) take on an intermediary position between that of Rat2 cells and A549 cells as shown in Fig. S2*A*. In the subsequent experiment, the time courses of HHS-CCN5(III)–mediated inhibition of phospho-AKT and phospho-ERK1/2 activities were analyzed. As shown in Fig. S2, *C* and *D*, HHS-CCN5(III) elicited rapid reduction of phospho-AKT (Ser473) and phospho-ERK1/2 (Thr202/Tyr204, Thr185/Tyr187) levels with maximal inhibition already after 60 min. Then, A549 cells were stimulated with increasing concentrations of recombinant HHS-CCN5(FL), HHS-CCN5(III), or HHS fusion partner alone for 60 min, and subjected to determination of cellular phospho-AKT (Ser473) levels by a time-resolved FRET–based immunoassay. As shown by the concentration-effect curves plotted in Figure 1*A*, full-length CCN5 (HHS-CCN5(FL)) and the TSP1 domain of CCN5 (HHS-CCN5(III)) reduced phospho-AKT (Ser473) levels in A549 cells with similar efficacy and potency (IC_50_ 25.1 μg/ml and 45.3 μg/ml with 95% Confidence Intervals 18.5–33.9 μg/ml and 18.2–112.9 μg/ml for HHS-CCN5(III) and HHS-CCN5(FL), respectively), whereas the HHS fusion partner alone did not display any efficacy. The data demonstrates that the TSP1 domain is sufficient to engender the intracellular signaling responses of CCN5. To corroborate the evidence of the TSP1 domain as the biologically active signaling peptide of CCN5, we also made the recombinant TSP1 domain as fusion protein with human albumin. Briefly, a fusion protein with signal peptide for secretion at the amino-terminal end and human albumin appended at the carboxyl-terminal end of the TSP1 domain (Alb-CCN5(III)) was expressed in CHO cells and purified from the cell culture media by sequential affinity chromatography and size-exclusion chromatography. Based on the extraordinary high degree of structural conservation of TSP1 homology domains among CCN proteins and report of the crystal structure of the TSP1 domain of CCN3 (8), we also made recombinant fusion protein of the TSP1 domain of CCN3 (Alb-CCN3(III)) configured similar to Alb-CCN5(III). As shown by the concentration-effect studies in Figure 1*B*, Alb-CCN5(III) and Alb-CCN3(III) reduced phospho-AKT (Ser473) levels in A549 cells with similar efficacy and potency (IC_50_ 15.3 μg/ml and 6.8 μg/ml with 95% Confidence Intervals: 2.8–81.8 μg/ml and 4.2–10.8 μg/ml for Alb-CCN5(III) and Alb-CCN3(III), respectively). Thus, our data demonstrate that the TSP1 domain of a CCN protein has the capacity to reduce phospho-AKT (Ser473) levels in A549 cells irrespective of the CCN protein from which it originates. Although the CCN(III) domains with albumin fusion partner appeared to be somewhat more potent than the ones generated with the HHS fusion partner (IC_50_ Alb-CCN5(III) 15.3 μg/ml *versus* HHS-CCN5(III) 25.1 μg/ml with 95% Confidence Intervals 2.8–81.8 μg/ml and 18.5–33.9 μg/ml, respectively), the TSP1 domains of CCN5 reduced phospho-AKT (Ser473) levels irrespective of the fusion partner. We subsequently investigated to what extent the TSP1 domain of CCN5 and CCN3 confers other signaling and biologic functions previously assigned to full-length CCN5. In order to assess the efficacy of the TSP1 domains of CCN5 and CCN3 at inhibiting CCN2-stimulated cell migration, we first investigated the concentration-effect relationships of CCN2-stimulated cell migration of Rat2 fibroblasts, as CCN2 has previously been reported to stimulate migration of Rat2 fibroblasts (10). As shown in Fig. S3*A*, the biologically active fragment of CCN2 (CCN2(III-IV)) dose-dependently stimulated the migration of Rat2 fibroblasts with EC_50_ approximately 13 μg/ml. In order to assess the efficacy of the recombinant TSP1 domain peptides at inhibiting CCN2-stimulated cell migration, Rat2 fibroblasts were stimulated with CCN2(III-IV) (5 μg/ml) and increasing concentrations of HHS-CCN5(III) and Alb-CCN3(III). As shown in Figure 1, *C* and *D*, HHS-CCN5(III) and Alb-CCN3(III) concentration-dependently inhibited CCN2-stimulated migration of Rat2 fibroblasts, whereas similar range of concentrations of the HHS fusion partner alone did not display efficacy at CCN2-stimulated cell migration (the X-axis uses μg/ml as units of concentration, however, the molar concentrations of HHS-CCN5(III) and HHS alone are not substantially different since the TSP1 domain is only about 5 kDa and a minor fraction of the total mass). Again, Alb-CCN3(III) tended to be slightly more potent than HHS-CCN5(III) (IC_50_ 6.4 μg/ml and 26.8 μg/ml with 95% Confidence Intervals 4.6–8.8 μg/ml and 24.7–29.1 μg/ml for Alb-CCN3(III)– and HHS-CCN5(III)–treated fibroblasts, respectively).

### The TSP1 domains of CCN5 and CCN3 inhibit CCN2-induced migration of fibroblasts

**Figure 2:**
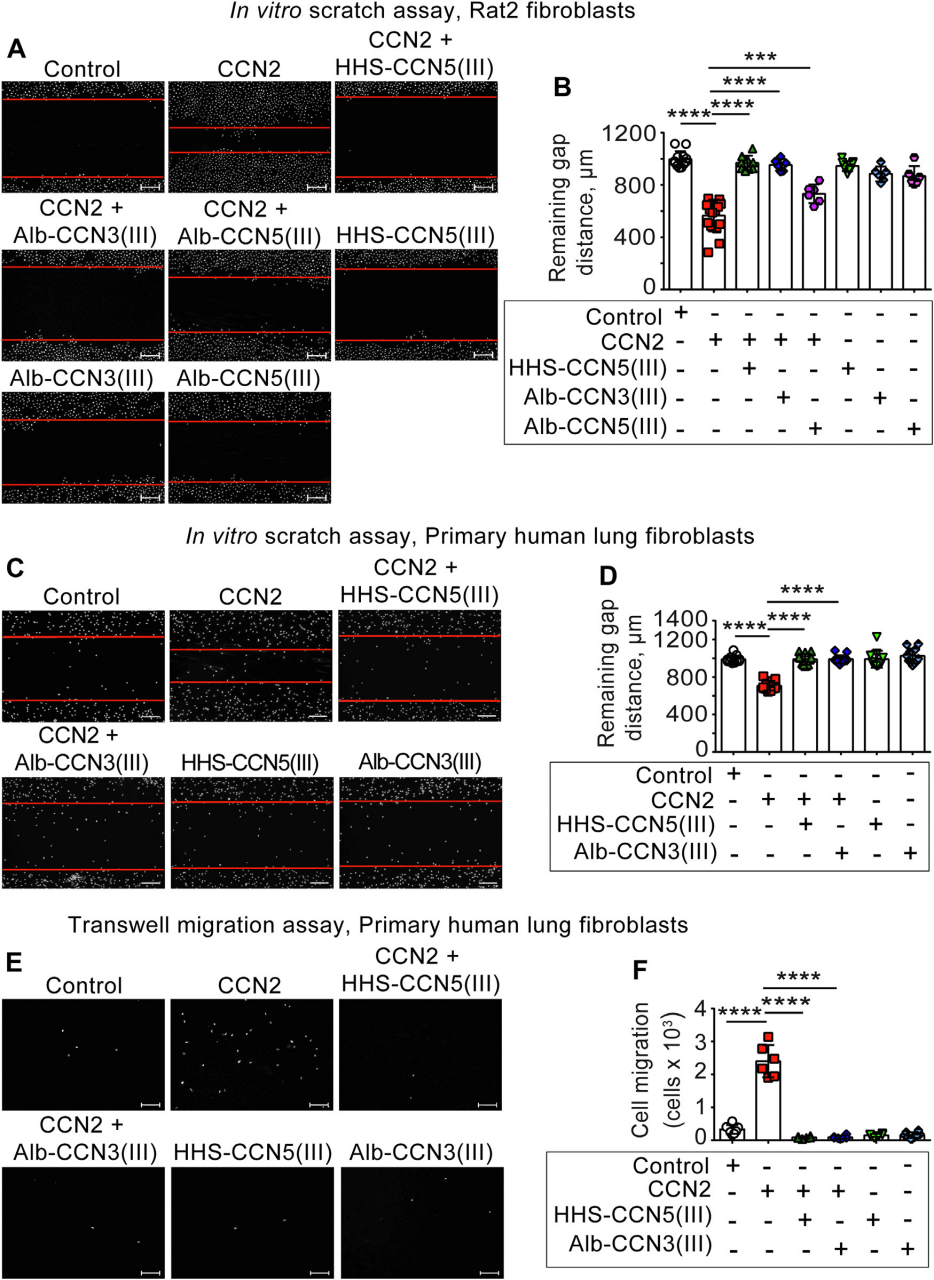
**The TSP1 domains of CCN5 and CCN3 inhibit CCN2-induced wound healing/transwell migration in Rat2 fibroblasts and primary human lung fibroblasts.** Panel (*A*) shows representative photomicrographs of *in vitro* wound healing assay (scratch assay) of the monolayer of Rat2 fibroblasts stimulated in the absence or presence of CCN2 (5 μg/ml) and HHS-CCN5(III) (100 μg/ml), Alb-CCN3(III) (60 μg/ml), or Alb-CCN5(III) (60 μg/ml) for 24 h following scratch wound. The cells were stained with fluorescent Hoechst dye 33258 before quantitative analysis of gap distances. Scale bar represents 200 μm. Scatter plot of gap distances is shown in Panel (*B*). The data represent the mean ± SD (n ≥ 3 independent experiments assayed in duplicate for each condition). Statistical significance was assessed by one-way ANOVA with Šidák’s post hoc test. ∗∗∗*p* < 0.001; ∗∗∗∗*p* < 0.0001 *versus* CCN2 group. Panel (*C*) demonstrates representative photomicrographs of *in vitro* wound healing assay (scratch assay) of monolayer of primary human lung fibroblasts stimulated in the absence or presence of CCN2 (5 μg/ml) and HHS-CCN5(III) (100 μg/ml), Alb-CCN3(III) (60 μg/ml), or Alb-CCN5(III) (60 μg/ml) for 24 h. Following 24 h of incubation, the cells were stained with Hoechst dye 33258 and the gap distances were quantified. Scale bar represents 200 μm. Scatter plot of gap distances is shown in Panel (*D*). The data represent the mean ± SD (n = 4 independent experiments assayed in duplicate for each condition). Statistical significance was calculated by one-way ANOVA with Šidák’s post hoc test. ∗∗∗*p* < 0.001; ∗∗∗∗*p* < 0.0001 *versus* CCN2 group. Panel (*E*) shows representative photomicrographs of transwell migration assay (Boyden chamber principle) of primary human lung fibroblasts stimulated in the absence or presence of CCN2 (5 μg/ml) and HHS-CCN5(III) (100 μg/ml), Alb-CCN3(III) (60 μg/ml), or Alb-CCN5(III) (60 μg/ml) for 20 h. Scale bar represents 100 μm. The cells that had migrated through the semi-permeable membrane were stained with Hoechst dye 33258 and counted. Panel (*F*) shows scatter plot of cell migration. The data represent the mean ± SD (n = 3 independent experiments assayed in duplicate for each condition). The data were subjected to statistical analysis by one-way ANOVA with Šidák’s post hoc test. ∗∗∗∗*p* < 0.0001 *versus* CCN2 group. Alb, albumin; CCN, Cellular Communication Network; HHS, His-Halo-Sumo; TSP1, thrombospondin type 1 repeat.

CCN2 has been reported to be essential to normal fibroblast functions, for example such as cell migration and fibrogenesis (14). As *in vivo* experimental studies have reported that overexpression of CCN5 may inhibit fibrosis (12), we used *in vitro* cell migration and scratch healing assays to investigate to what extent the TSP1 domain of CCN5 would be sufficient to confer the biologic functions of CCN5. The Rat2 fibroblast is an embryonic fibroblast cell line that responds particularly well to exogenous stimulation with recombinant CCN2 (10, 27). Thus, we first assessed HHS-CCN5(III)– and Alb-CCN5(III)–mediated inhibition of CCN2-stimulated *in vitro* wound healing (*in vitro* scratch assay) of Rat2 fibroblasts. We also investigated Alb-CCN3(III)-mediated inhibition of CCN2-stimulated *in vitro* wound healing in order to uncover to what extent the TSP1 domain of CCN3 would support similar actions. As shown in Figure 2, *A* and *B*, all TSP1 fusion proteins abrogated or significantly inhibited CCN2-stimulated closure of the wound gap (gap closure). Thus, the TSP1 domain of CCN5 confers the actions of full-length CCN5 also for the more complex biological response of *in vitro* wound healing. As shown in Figure 2, *C* and *D*, the TSP1 domain of CCN5 and CCN3 also inhibited CCN2-stimulated gap closure in *in vitro* assay of wound healing of primary human lung fibroblasts. Primary human lung fibroblasts were found to respond to CCN2 with increased phospho-AKT (Ser473) levels similar to Rat2 fibroblasts (E_max_ approximately 50 folds stimulation relative to unstimulated cells and EC_50_ 18.4 μg/ml (with 95% Confidence Intervals 8.3–40.7 μg/ml)). In congruence with these findings, the TSP1 domain of both CCN5 and CCN3 robustly inhibited CCN2-stimulated transwell migration of both Rat2 fibroblasts (Fig. S3, *B* and *C*) and primary human lung fibroblasts (Fig. 2, *E* and *F*). Thus, the findings reveal that the functions of the TSP1 domain of CCN5 and CCN3 transcend species and cell line boundaries. Despite limited evidence, CCN5 has been proposed to be a ubiquitous inhibitor of all four-domain CCN proteins (11, 28). Thus, we also investigated to what extent the TSP1 domain would be sufficient to inhibit CCN1-stimulated cell migration of Rat2 fibroblasts. Indeed, as shown in Fig. S3, *D* and *E*, the TSP1 domain of CCN3 inhibited CCN1-stimulated cell migration of Rat2 fibroblasts. Thus, the TSP1 domain of CCN proteins appears to confer broad inhibition of biologically active four-domain CCN protein isoforms.

### CCN2-induced cell migration is mediated *via* ERK1/2 activation in Rat2 fibroblasts

**Figure 3:**
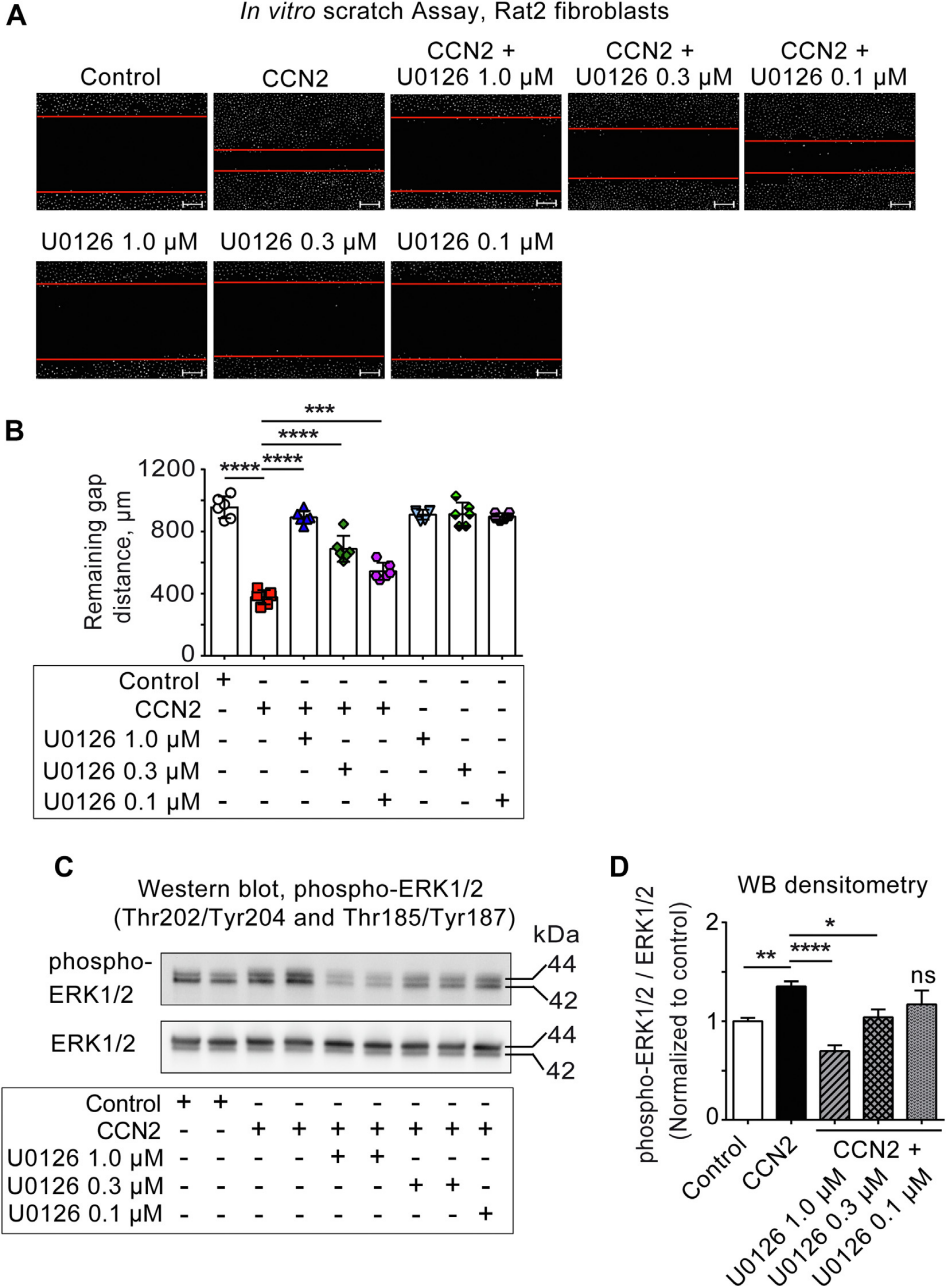
**CCN2-induced cell migration is mediated *via* ERK1/2 activation.** Panel (*A*) shows representative photomicrographs of *in vitro* scratch-wound assay of monolayer of Rat2 fibroblasts stimulated in the absence (vehicle control) or presence of CCN2 (5 μg/ml CCN2) or together with the increasing concentrations of U0126 (0.1–1.0 μM) for 24 h. U0126 was added to the cell culture medium 30 min prior to stimulation with CCN2. Following 24 h of incubation, the cells were stained with fluorescent Hoechst dye 33258 and the gap distances were recorded. Scale bar represents 200 μm. The scatter plot in panel (*B*) provides the recorded gap distances. The data represent the mean ± SD (n = 3 independent experiments assayed in duplicate for each condition). Panel (*C*) demonstrates representative photomicrograph of Western blot of cell lysates from Rat2 fibroblasts stimulated in the absence (vehicle control) or presence of CCN2 (5 μg/ml) or together with the increasing concentrations of U0126 (0.1–1.0 μM) for 24 h. Cells were stimulated with U0126, 30 min prior to stimulation with CCN2. The samples were separated by SDS gel electrophoresis, transferred to PVDF membranes, and immunoblotted with antibodies against phospho-ERK1/2 (Thr202/Tyr204 and Thr185/Tyr187) and total ERK1/2 (loading control). The photomicrograph demonstrates the immunoreactive bands and is representative of two independent experiments with two replicates per condition. Panel (*D*) demonstrates histogram of data following densitometric analysis of immunoreactive bands and shows phospho-ERK1/2 levels relative to total ERK1/2 of the two independent experiments presented as mean ± SEM. Statistical significance was assessed by one-way ANOVA with Šidák’s post hoc test. ∗*p* < 0.05; ∗∗*p* < 0.01; ∗∗∗*p* < 0.001; ∗∗∗∗*p* < 0.0001 *versus* CCN2 group. Uncropped immunoblots are shown in Fig. S4, *A* and *B*. CCN, Cellular Communication Network.

Current evidence indicates that propagating waves of ERK1/2 activity is critical for directional cell migration (29, 30) and it has been suggested that CCN2-induced migration may depend on ERK1/2-signaling (14). Thus, we first investigated CCN2-stimulated time course of AKT and ERK1/2 phosphokinase activation within a time span relevant for CCN2-stimulated migration of Rat2 fibroblasts. As shown in Fig. S2, *E* and *F*, both phospho-AKT (Ser473) and phospho-ERK1/2 (Thr202/Tyr204 and Thr185/Tyr187) levels rise rapidly after stimulation with CCN2. Both phosphokinases subsequently display fast decay towards basal levels. Interestingly, whereas phospho-AKT levels remain at basal levels at 24 h following stimulation with CCN2, phospho-ERK1/2 levels display secondary peak activities around 24 h, indicative of oscillatory-like activity according to previous reports (31, 32). In other cell types, delayed or sustained stimulation of phospho-ERK levels have also been reported (33). Thus, in order to provide experimental evidence for a key role of ERK1/2 in CCN2-induced healing of *in vitro* scratch-wound and cell migration, Rat2 fibroblasts were subjected to *in vitro* scratch assay in the presence or absence of CCN2 and/or U0126 (a selective MEK1/MEK2 inhibitor) (Fig. 3, *A* and *B*) and analyzed for gap closure following 24 h of stimulation. Increasing concentrations of U0126 significantly inhibited CCN2-stimulated closure of the scratch wound (gap closure), indicating that ERK1/2 plays a major role in CCN2-induced scratch-wound closure and cell migration in these cells. Western blot analysis of the cell extracts also revealed that U0126 inhibited CCN2-stimulated phosphorylation of ERK1/2 (Thr202/Tyr204 and Thr185/Tyr187) in a concentration-dependent manner, suggesting the involvement of ERK1/2 phosphorylation in cell migration and gap closure of *in vitro* scratch wound (Fig. 3, *C* and *D*).

### The TSP1 domains of CCN5 and CCN3 inhibit CCN2-induced cell migration by affecting ERK1/2 signaling

**Figure 4:**
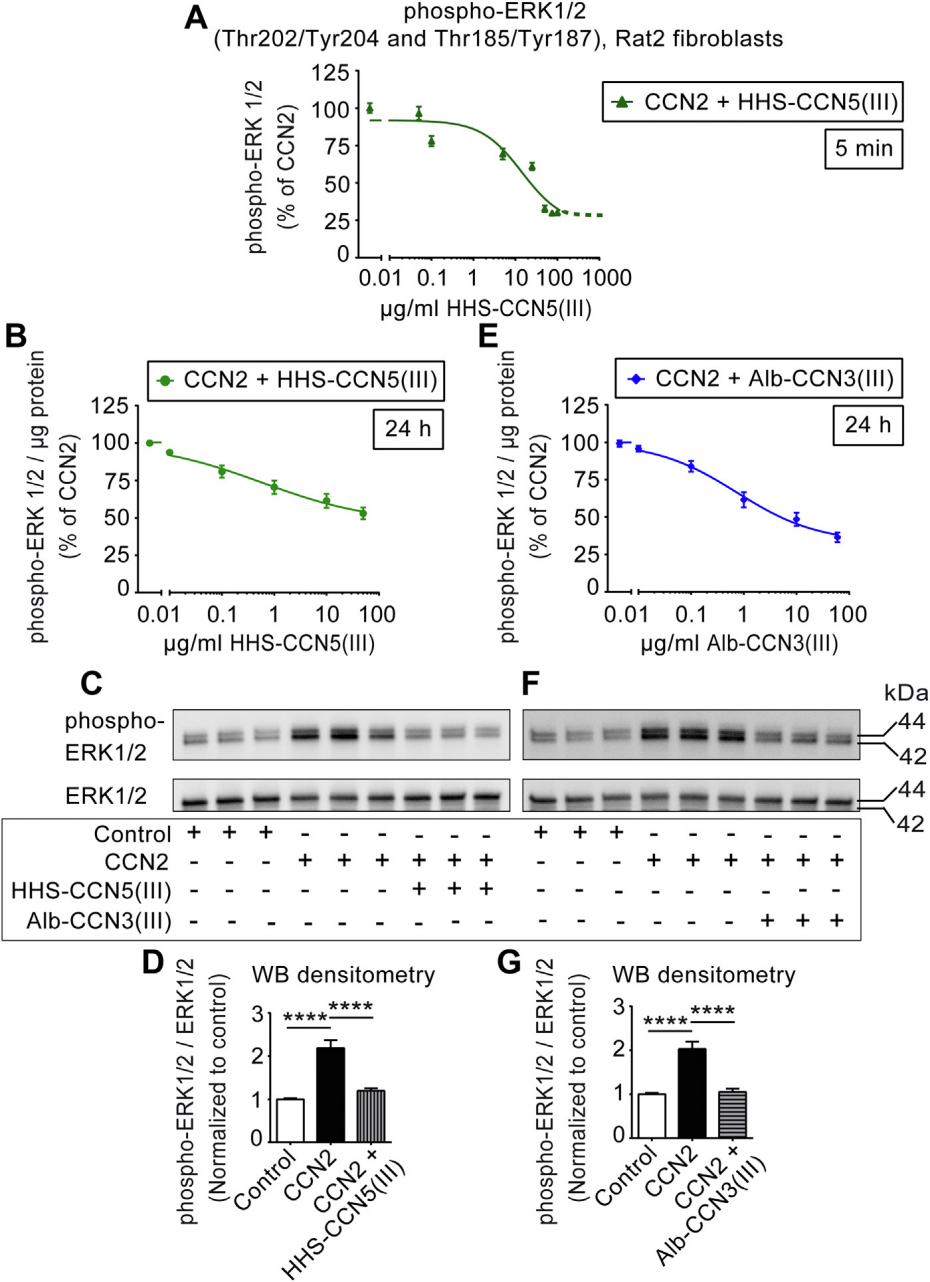
**The TSP1 domains of CCN5 and CCN3 inhibit CCN2-stimulated phospho-ERK1/2 activities and CCN2-induced cell migration.** Panels (*A*) and (*B*) show concentration-effect curves of HHS-CCN5(III)–engendered inhibition of CCN2-stimulated phospho-ERK1/2 activities in Rat2 fibroblasts following exposure to CCN2 and increasing concentrations of HHS-CCN5(III) for 5 min or 24 h, respectively. Panel *E* demonstrates concentration-effect curve of Alb-CCN3(III)–engendered inhibition of CCN2-stimulated phospho-ERK1/2 activities in Rat2 fibroblasts following 24 h exposure to CCN2 and increasing concentrations of Alb-CCN3(III). Total cell lysates of Rat2 fibroblasts stimulated for 5 min or 24 h in the absence (vehicle control) or presence of CCN2 (5 μg/ml CCN2) alone or together with increasing concentrations of HHS-CCN5(III) or Alb-CCN3(III) were analyzed by HTRF-based (PerkinElmer) or Luminex-based immunoassay (BioPlex) of phospho-ERK1/2 (Thr202/Tyr204 and Thr185/Tyr187) contents. Data are phospho-ERK1/2 levels relative to total protein content of cell lysate presented as percent of CCN2-stimulated activities. The data represent the mean ± SEM (n = 3 independent experiments assayed in 3–4 replicates for each data point). The data in Panels (*A*), (*B*), and (*E*) were subjected to curve fitting by four-variable nonlinear regression in GraphPad Prism. Panels (*C*) and (*F*) demonstrate representative photomicrographs of Western blot analysis of cell lysates of Rat2 fibroblasts stimulated for 24 h in the absence (vehicle control) or presence of CCN2 (5 μg/ml) alone or as coincubation with HHS-CCN5(III) (50 μg/ml) or Alb-CCN3(III) (60 μg/ml). The cell lysates were separated by SDS gel electrophoresis, transferred to PVDF membranes, and immunoblotted with antibodies against phospho-ERK1/2 (Thr202/Tyr204 and Thr185/Tyr187) and total ERK1/2 (loading control). The photomicrographs (*C* and *F*) demonstrate the immunoreactive bands and are representative of three independent experiments with three replicates per condition. Panels (*D*) and (*G*) show histograms of densitometric analysis of phospho-ERK1/2 immunoreactivities relative to total ERK1/2 immunoreactivities. The data represent the mean ± SEM (n = 3 independent experiments assayed in three replicates for each condition). Statistical significance was assessed by one-way ANOVA with Šidák’s post hoc test. ∗∗∗∗*p* < 0.0001 *versus* CCN2 group. Alb, albumin; CCN, Cellular Communication Network; HHS, His-Halo-Sumo; HTRF, homogenous time-resolved fluorescence; TSP1, thrombospondin type 1 repeat.

To further assess the inhibitory effect of the TSP1 domain of CCN5 on CCN2-stimulated ERK1/2 activation, nonconfluent Rat2 fibroblasts were stimulated with CCN2 and increasing concentrations of fusion protein containing the TSP1 domain of CCN5 (HHS-CCN5(III)) for both short-term exposure (5 min) and long-term exposure (24 h). Cell extracts were subsequently subjected to either HTRF (homogenous time-resolved fluorescence)-based (PerkinElmer) or Luminex bead-based immunoassay (BioPlex) of phospho-ERK1/2 levels. As shown in Figure 4, *A* and *B*, HHS-CCN5(III) dose-dependently inhibited CCN2-stimulated phospho-ERK1/2 (Thr202/Tyr204 and Thr185/Tyr187) levels in Rat2 fibroblasts both following 5 min or 24 h exposures with IC_50_ values for HHS-CCN5(III) of approximately 14 μg/ml (with 95% Confidence Intervals 8.1–25.4 μg/ml) or 0.5 μg/ml (with 95% Confidence Intervals 0.03–10.28 μg/ml), respectively. Nonconfluent Rat2 fibroblasts stimulated with CCN2 in the absence or presence of the TSP1 domain of CCN5 (HHS-CCN5(III)) for 24 h were also subjected to Western blot analysis of phospho-ERK1/2 (Thr202/Tyr204 and Thr185/Tyr187) activities. As shown in Figure 4, *C* and *D*, the intensities of immunoreactive phospho-ERK1/2 in CCN2-stimulated cells were inhibited by HHS-CCN5(III). Similarly, nonconfluent Rat2 cells were subsequently stimulated with CCN2 and increasing concentrations of the TSP1 domain of CCN3 with albumin as fusion partner (Alb-CCN3(III)) and subjected to Luminex bead-based immunoassay of cellular phospho-ERK1/2 contents. As shown in Figure 4*E*, Alb-CCN3(III) also dose-dependently inhibited CCN2-stimulated phospho-ERK1/2 levels with IC_50_ approximately 0.7 μg/ml. Furthermore, Western blot analysis of Rat2 cells stimulated in the absence or presence of CCN2 and Alb-CCN3(III) also showed that the TSP1 domain of CCN3 was able to abolish the CCN2-stimulated increase of cellular phospho-ERK1/2 levels (Figure 4, *F* and *G*). Fig. S3*F* shows histogram of the immunoassay of phospho-ERK1/2 (Thr202/Tyr204 and Thr185/Tyr187) activities following stimulation of Rat2 fibroblasts in the presence or absence of CCN2, HHS-CCN5(III), or Alb-CCN3(III). The histogram demonstrates that the TSP1 domains of CCN5 or CCN3 are both able to inhibit basal, unstimulated phospho-ERK1/2 activities in Rat2 fibroblasts. Thus, the TSP1 domain of CCN5 is a biologically active peptide that appears sufficient in conferring the inhibitory effect of CCN5 at a major signaling pathway involved in cell migration. Corroborating the capacity of the homologous TSP1 domain of CCN3 to recapitulate the effects of the TSP1 domain of CCN5, this study also demonstrates that the TSP1 domain of CCN3 (Alb-CCN3(III)) inhibits CCN2-stimulated phospho-ERK1/2 activities with similar efficacy and potency as that of the TSP1 domain of CCN5 (HHS-CCN5(III)) (Fig. 4, *B* and *E*).

### The TSP1 domain of CCN5 inhibits CCN2-stimulated collagen gel contraction

**Figure 5:**
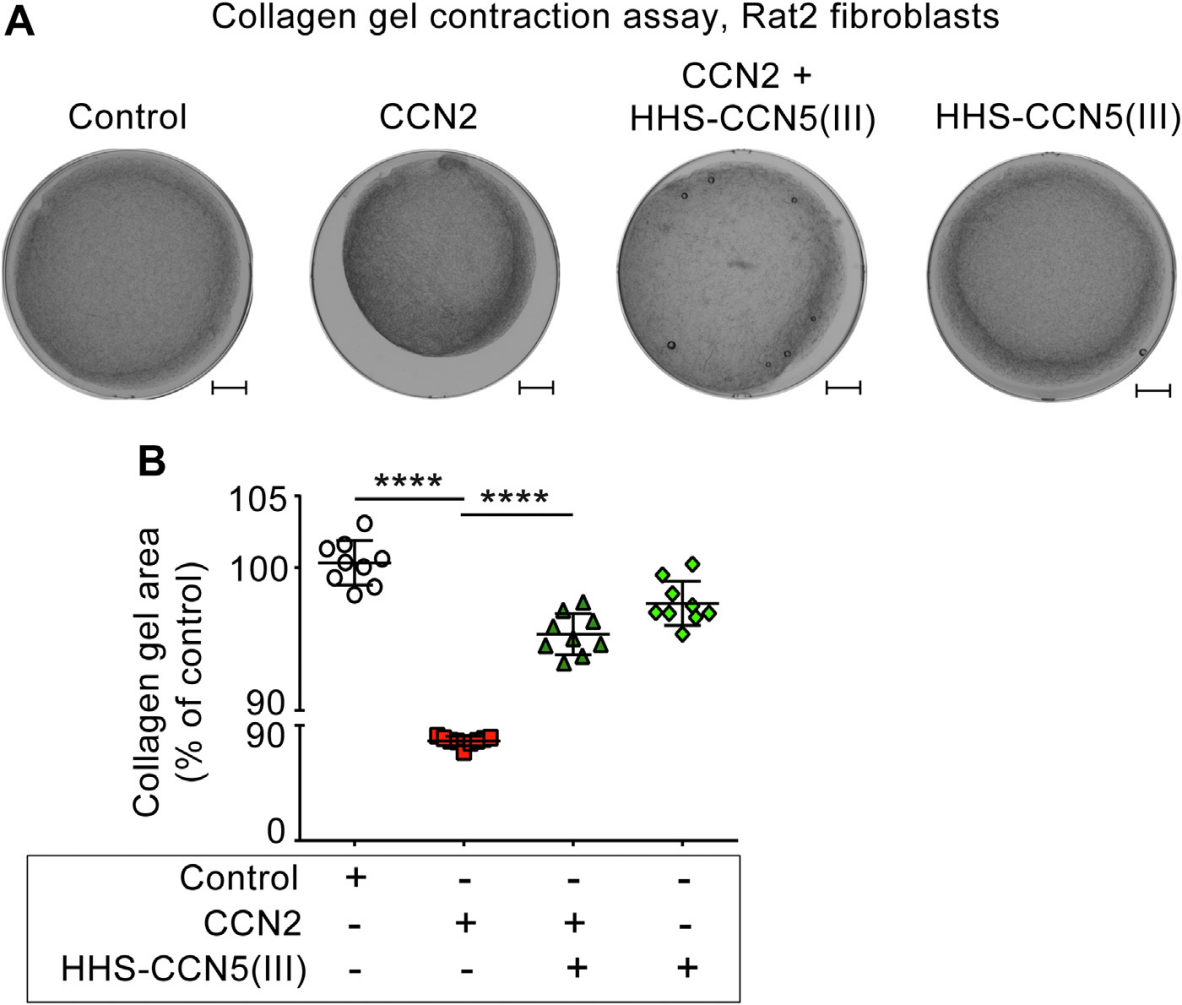
**The TSP1 domain of CCN5 inhibits CCN2-induced collagen gel contraction.** The figure demonstrates representative photographs (*A*) of collagen gel contraction assay of Rat2 fibroblasts stimulated in the absence or presence of CCN2 (20 μg/ml), HHS-CCN5(III) (200 μg/ml), or CCN2 (20 μg/ml) + HHS-CCN5(III) (200 μg/ml). Scale bar represents 2 mm. Panel (*B*) shows scatter plot of quantitative analysis of gel contraction assay. The results represent the mean ± SD (n = 3 independent experiments assayed in three replicates per condition). Statistical significance was assessed by one-way ANOVA with Šidák’s post hoc test. ∗∗∗∗*p* < 0.0001 *versus* CCN2 group. CCN, Cellular Communication Network; HHS, His-Halo-Sumo; TSP1, thrombospondin type 1 repeat.

The collagen lattice contraction assay provides a model for myofibroblast-mediated scar tissue contraction that allows for investigations of the influence of specific substances on the rate and extent of matrix contraction. Rat2 fibroblasts were stimulated with or without CCN2 in the absence or presence of HHS-CCN5(III) in a solidified collagen gel matrix and incubated for 4 days in a cell incubator. As shown in Figure 5, *A* and *B*, CCN2-stimulated gel contraction was substantially inhibited by the TSP1 domain of CCN5 (HHS-CCN5(III)).

### The TSP1 domain of CCN5 inhibits CCN2-induced mammosphere formation of mammary adenocarcinoma cells and induces markers of cell differentiation

**Figure 6:**
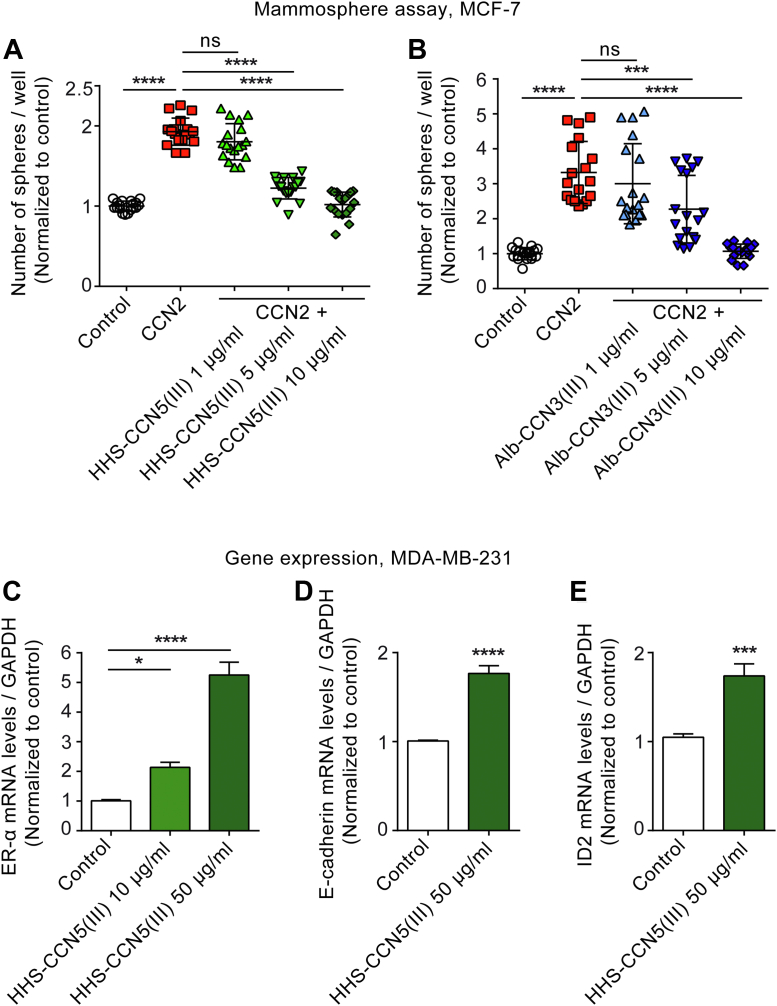
**The TSP1 domain of CCN5 inhibits CCN2-induced mammosphere formation in MCF-7 cells and induces ER-α, E-cadherin, and ID2 mRNAs in MDA-MB-231 cells.** Single-cell suspensions of MCF-7 mammary carcinoma cells were seeded in ultra-low adhesion plates and cultured under mammosphere-forming conditions for 7 days in the absence (vehicle control) or presence of CCN2 (1 μg/ml) and HHS-CCN5(III) or Alb-CCN3(III) (1, 5, or 10 μg/ml). The cells were subsequently stained for cell viability with the tetrazolium dye MTT, and the spheres were semiautomatically quantified with 40 μm cut-off diameter. Panels (*A*) and (*B*) demonstrate data for stimulation with CCN2 and coincubation in the presence of HHS-CCN5(III) or Alb-CCN3(III), respectively. The data represent the mean ± SD (n = 3 independent experiments assayed in six replicates for each condition). Statistical significance was assessed by one-way ANOVA with Šidák’s post hoc test. ∗∗∗*p* < 0.001; ∗∗∗∗*p* < 0.0001; ns: indicates not statistically significant *versus* CCN2 group. Panels (*C*), (*D*), and (*E*) show histograms of mRNA levels of ER-α, E-cadherin, and ID2 relative to GAPDH in MDA-MB-231 2D cell cultures, serum-starved in 0.1% serum overnight, and stimulated with (HHS(CCN5(III) (10 or 50 μg/ml) for 48 h. mRNA levels were determined by real-time qPCR using TaqMan probes. The results were normalized to vehicle control and represent the mean ± SEM (n = 4 independent experiments assayed in duplicate for each condition). Statistical significance for panel (*C*) was assessed by one-way ANOVA with Dunnett’s post hoc test. ∗*p* < 0.05; ∗∗∗∗*p* < 0.0001 *versus* control group. Statistical significance for panels (*D* and *E*) was assessed by unpaired Student’s two tailed *t* test. ∗∗∗*p* < 0.001; ∗∗∗∗*p* < 0.0001 *versus* control group. Alb, Albumin; CCN, Cellular Communication Network; ER-α, estrogen-receptor α; HHS, His-Halo-Sumo; TSP1, thrombospondin type 1 repeat.

We have previously reported that biologically active CCN2 stimulates mammosphere formation of MCF-7 mammary carcinoma cells under anchorage-independent growth conditions (10). In order to obtain broader evidence for the TSP1 domain of CCN5 being the biologically active entity sufficient for antagonizing CCN2-stimulated functions, we investigated to what extent the recombinant TSP1 domains of CCN5 and CCN3 were able to inhibit CCN2-induced mammosphere formation of MCF-7 cells. As shown in Figure 6, *A* and *B*, the TSP1 domain of both CCN5 (HHS-CCN5(III)) and CCN3 (Alb-CCN3(III)) inhibited CCN2-induced sphere formation of MCF-7 cells in a concentration-dependent manner. These findings demonstrate that the TSP1 domains of CCN5 and CCN3 are bioactive peptides that are capable of replicating yet another function previously assigned to full-length CCN5 (34). Inhibition of mammosphere formation of mammary adenocarcinoma cells is associated with the inhibition of stem cell features and epithelial-to-mesenchymal transition (EMT). Full-length CCN5 has previously been reported to restore responsiveness of triple negative MDA-MB-231 mammary adenocarcinoma cells to estrogens by induction of ER-α mRNA (35). Thus, we investigated to what extent treatment of MDA-MB-231 cells with the TSP1 domain of CCN5 would induce ER-α mRNA as well as mRNA markers of cell differentiation and reversal of EMT (36). As shown in Figure 6, *C*–*E*, TSP1 domain of CCN5 induced robust increase of ER-α mRNA levels as well as of mRNA levels of the differentiation markers E-cadherin and ID2 in MDA-MB-231 cells, indicating that the TSP1 domain of CCN5 may promote differentiation of mammary adenocarcinoma cells towards a less aggressive and invasive phenotype by inhibiting EMT and inducing responsiveness to estrogens.

## Discussion

In this study, we report the novel finding that the TSP1 homology domain of CCN5 is a biologically active signaling entity that exerts the functions previously assigned to full-length CCN5. We find that the TSP1 domain of CCN5 rapidly inhibits key signaling pathways previously reported to be stimulated by CCN1, CCN2, and CCN3, that is, the AKT phosphokinase and ERK1/2 phosphokinase pathways as well as CCN1/2-stimulated cell migration and gap closure following *in vitro* scratch wound (10). The TSP1 homology domain of CCN3 exerted these functions with similar efficacy and potency as that of the TSP1 domain of CCN5. The TSP1 domain of CCN5 also recapitulated a positive regulatory function previously assigned to full-length CCN5, that is, induction of ER-α expression in triple negative MDA-MB-231 mammary adenocarcinoma cells.

The variable and unstructured hinge region of the CCN proteins located between the vWC homology domain and the TSP1 homology domain has previously been reported to be sensitive to various endopeptidases including several of the MMPs (6, 37). The function of such a region hypersensitive to several endopeptidases has remained elusive. However, we recently reported that CCN2 is a secreted preproprotein that requires proteolytic activation in order to release its biologically active signaling entity (10). Indeed, proteolytic activation of CCN2 entailed cleavage of MMP-sensitive sites in the hinge region, leading to release of the biologically active carboxyl-terminal fragment consisting of the TSP1-homology domain and the CK domain.

Proteolytic activation in order to release a biologically active signaling entity is certainly not unique to CCN2. Synthesis of preproproteins that need to undergo proteolytic activation in order to release the biologically active entities is a way of controlling the activities of many secreted proteins, including hormones and autocrine/paracrine factors. In this context, other matricellular proteins have also been reported to undergo proteolytic cleavage in order to release biologically active signaling entities, for example, SPARC, TGF-β, and Hevin (38, 39, 40). We also demonstrated that the carboxyl-terminal fragments of CCN1 and CCN3 consisting of the TSP1 and CK domains are fully active biological entities (10). However, to what extent full-length CCN1 and CCN3 may be regarded as latent proforms that require proteolytic activation still remains to be resolved. Full-length CCN1 and CCN3 proved to be particularly sensitive to proteolytic cleavage at the cell surface (6, 10). Thus, activities revealed by stimulation with the full-length proteins may well be due to instant proteolytic activation. Butler *et al.* (6) reported that the hinge region of CCN5 was a substrate of MMP14, a membrane-bound metalloproteinase. However, this MMP isoform was also found to cleave the hinge region of several of the other CCN proteins, including CCN1, CCN2, and CCN3 (6). Upon investigation of what fragment of CCN5 conferred its antiangiogenic properties, the authors found that the antiangiogenic activity was located in the carboxyl-terminal TSP1 domain and not in the N-terminal fragment of CCN5. Yet, the authors did not conclude that the TSP1 domain of CCN5 was the biologically active entity. Rather, based on their observation that overexpression of MMP14 abrogated the antiangiogenic activity of CCN5, they concluded that cleavage of CCN5 might cause inactivation of CCN5. However, based on the finding that other CCN proteins might also be activated by proteolytic cleavage, an alternative interpretation could as well be that overexpression of MMP14 might activate proangiogenic CCN1 and CCN2 and thus mask the activity of CCN5. In this context, small peptides derived from the TSP1 domain of CCN5, as well as other CCN protein isoforms, have been reported to inhibit endothelial cell proliferation and migration (41). However, such synthetic peptides from the TSP1 domain lacking tertiary structure appear to be ubiquitous CCN protein inhibitors (41).

Min Ho Song *et al.* (42) recently reported that the TSP1 domain of CCN5 was essential and sufficient for endocytosis and nuclear localization of CCN5, but the TSP1 domain was not able alone to confer reverse trans-differentiation of myofibroblasts to fibroblasts. The authors concluded that either the insulin-like growth factor–binding protein domain or the vWC domain of CCN5 was required in addition to the TSP1 domain to exert the latter function (42). It is difficult to reconcile how two such structurally different domains can rescue the same functional effects. Based on the complex disulfide connectivity of the TSP1 domain, poor expression in recombinant systems, and poor solubility, our data point to the fusion partner as a chaperone that supports proper folding, expression, and solubility of the TSP1 domain. The various CCN(TSP1) fusion proteins used in this study consistently exerted similar functions and inhibited CCN2-stimulated activities with similar efficacy and potency. Thus, the fusion partners acted as a good internal control for aberrant activities not related to the TSP1 domain of CCN5. In addition, the HHS fusion partner alone did not display activity in phosphokinase and cell migration assays corroborating the evidence assigned to the TSP1 domain of CCN5. The TSP1 domain of CCN5 appended to human albumin may also open up for *in vivo* physiologic studies of the functions of CCN5, as the TSP1 domain by itself is small (approx. 5 kDa) and can be expected to be rapidly excreted following parenteral administration. In this respect, albumin is a recognized fusion partner in biologic drug design for pharmacokinetic modulation (43).

The mechanism by which the TSP1 domain of CCN5 (or that of other CCN proteins) inhibits functions of bioactive entities of four-domain CCN proteins (*e.g.*, CCN1 or CCN2) still remains obscure. Two modes of action may theoretically be envisaged. The TSP1 domain of CCN5 may either bind and neutralize other stimulatory CCN proteins or exert antagonist function by competing at potential receptor-binding sites. The carboxyl-terminal CK domain present in four-domain CCN proteins have previously been reported to facilitate homo-dimer formation in congruence with carboxyl-terminal CK domains in a number of other proteins including growth factors, autocrine/paracrine factors, and certain cytokines (2, 44, 45, 46). Indeed, we have previously demonstrated that bioactive CCN2 can exist as a homo-dimer of the carboxyl-terminal fragment consisting of the TSP1 and the CK domains (10). Furthermore, this homo-dimer of the carboxyl-terminal fragment of CCN2 was found to be substantially more potent than the monomeric fragment (10). Yet, homo-dimer formation of CCN2(III-IV) (also called d-3-4-CCN2) during recombinant protein production was not stoichiometric and only entailed a small fraction of the protein produced (10). To what extent CCN5 lacking the carboxyl-terminal CK domain may engage in homo-dimer formation, or indeed, may heterodimerize with other CCN proteins, remains to be demonstrated. In any case, we have not observed homo-dimer formation of any of the fusion proteins with the TSP1 domain of CCN5 produced in recombinant expression systems. Thus, based on current evidence, it appears more plausible that the TSP1 domain of CCN5 (or that of other CCN proteins) inhibits bioactive entities of four-domain CCN proteins by acting as an antagonist competing for putative receptor-binding sites.

TSP1 domains, also previously known as thrombospondin type 1 repeats, are one of the most common motifs found in extracellular proteins (47). TSP1 domains have been reported to exert functions through binding of many different membrane proteins or through binding of other proteins in the extracellular matrix. Among documented binding interactions are integrins, CD36, latent TGF-β, VEGF (vascular endothelial growth factor), collagen, fibronectin, and heparin sulfate–containing proteoglycans (8, 48, 49, 50, 51, 52, 53) (for review, see refs (54, 55)). As to CCN proteins, the TSP1 domain of CCN2 has been reported to bind VEGF^165^ and sequester VEGF^165^ from its receptors (52). However, cleavage of the hinge region of CCN2 between domains II and III was found to eliminate VEGF^165^ binding (52). Furthermore, the TSP1 domain of CCN1 has been found to bind integrin α_6_β_1_ (53). A question yet to be resolved is to what extent the TSP1 domain of CCN5 deviates from TSP1 domains of other protein families sufficient to confer distinct functions. First, although many TSP1 domains have been found to bind latent TGF-β, the TSP1 domain of CCN proteins lacks the KRFK peptide motif reported to be required for binding to TGF-β (48). Interestingly, the crystal structure recently reported for the TSP1 domain of CCN3 disclosed altered disulfide connectivity and lack of the typical π-stacked ladder of charged and aromatic amino acids present on one side of the domain that is found in TSP1 domains of other protein families (8). From this structure, it was concluded that the TSP1 domain of CCN3, and likely those of the other CCN family proteins, deviates from TSP1 domains of other protein families and thus forms a subfamily, suggesting that the TSP1 domain of CCN proteins may exert distinct functions. For this reason, and to be able to use the structure of CCN3 as a guide for putative modifications of the TSP1 domain of CCN5, we also prepared and investigated the recombinant TSP1 domain of CCN3 amino-terminally fused to human albumin. Interestingly, the TSP1 domain of CCN3 inhibited phosphokinase signaling and cell migration with similar potency and efficacy as albumin-CCN5(III). These findings are consistent with a previous report demonstrating that CCN2 lacking the carboxyl-terminal CK domain exerted similar antihypertrophic effects as CCN5 in primary cardiac myocytes stimulated with phenylephrine (13). Conversely, when the CK domain of CCN2 was appended to the carboxyl-terminus of CCN5, the modified CCN5 became functionally similar to CCN2. Thus, the TSP1 domain of various CCN proteins appears to exert similar functions. Yet, how lack of the CK domain makes CCN5 exert converse actions to those of the four-domain CCN protein isoforms remains to be resolved. In any case, pin-pointing inhibition of ERK1/2 as a target of CCN(TSP1) action may have important implications as ERK1/2 has been shown to be a key kinase involved in the stimulation of cell motility and collective cell migration, that is, both critical events in fibrogenesis and fibrosis (29, 30, 56). Furthermore, elevated levels of phospho-ERK1/2 have previously been shown to be present in tissue from fibrotic lungs and to normalize upon antifibrotic interventions (57), further highlighting ERK1/2 as a kinase involved in fibrosis. Thus, the TSP1 domain of CCN5 may facilitate the engineering of new therapeutic molecules that could potentially be effective as antifibrotic therapies or as anticancer drugs.

As many of the reported functions of CCN5 are found to be antagonistic to those of four-domain CCN proteins, we also investigated to what extent positive gene regulatory functions of CCN5 could be recapitulated by the TSP1 domain of CCN5. Full-length CCN5 has previously been reported to induce estrogen receptor ER-α mRNA in triple negative MDA-MB-231 mammary adenocarcinoma cells (35) and to inhibit EMT (36). In this study, we find that the TSP1 domain of CCN5 is sufficient in inducing ER-α mRNA and mRNA markers (E-cadherin and ID2) associated with the inhibition of EMT of MDA-MB-231 cells. These findings are also consistent with our observations that the TSP1 domains of both CCN5 and CCN3 inhibit CCN2-induced mammosphere formation of MCF-7 cells subjected to adhesion-independent growth conditions. Yet, how the TSP1 domain putatively acting as an antagonist of four-domain CCN proteins may induce gene expression of estrogen receptor ER-α mRNA remains elusive.

In conclusion, this study provides broad evidence from several signaling systems that the TSP1 domain is the biologically active signaling entity of CCN5. To what extent full-length CCN5 can be considered a latent proform, that is, cleavage of CCN5 in the variable hinge region is obligatory in order to become biologically active, similar to CCN2, remains to be resolved. In any case, delineation of the TSP1 domain of CCN5 as the biologically active signaling entity does not contradict previous data on CCN5. The various domains of CCN5 may still have specific roles in the compartmentation of CCN5 as well as in interactions with other matricellular proteins. Certainly, cleavage in the hinge region by specific endopeptidases may add an additional checkpoint for controlled release of bioactive CCN5 in specific pathophysiological conditions. Furthermore, delineation of the TSP1 domain as the biologically active signaling entity may facilitate studies on the functions of CCN5 and its potential therapeutic use in disease conditions.

## Experimental procedures

### Cell lines and reagents

Rat2 fibroblasts (CRL-1764, American Type Culture Collection (ATCC)) and MCF-7 (HTB-22, ATCC) and MDA-MB-231 (HTB-26, ATCC) human mammary adenocarcinoma cells were maintained in Dulbecco’s modified Eagle’s medium (DMEM) with high glucose (Gibco), supplemented with 10% fetal bovine serum (FBS) (Gibco) and 50 μg/ml gentamycin sulfate (Sanofi, Cat. No 453130). A549 human lung carcinoma cells (CRM-CCL-185, ATCC) were maintained in DMEM/F-12 with GlutaMAX (Gibco, Cat. No 31331028) supplemented with 10% FBS and 50 μg/ml gentamycin sulfate. Primary Human Lung Fibroblasts (NHLF) (Lonza, Cat. No CC-2512) were maintained in Fibroblast Growth Basal Medium (Lonza, Cat. No CC-3131), supplemented with FGM-2 Fibroblast Growth Medium-2 SingleQuots Supplements & Growth Factors (Lonza, Cat. No CC-4126). All experiments with primary human lung fibroblasts (NHLF) were carried out at early cell passage numbers (cell passage no. 2-5). U0126 (a selective inhibitor of MEK1 and MEK2) was purchased from TOCRIS. All other chemicals and reagents were analytical grade from Merck Life Sciences unless otherwise stated.

### Plasmid expression vectors

Ubiquitous chromatin opening elements-CMV plasmid expression vectors encoding fusion protein with full-length CCN5 (HHS-CCN5(FL)), fusion proteins with the TSP1 homology domain of CCN5 (HHS-CCN5(III) or Alb-CCN5(III)) or CCN3 (Alb-CCN3(III)), or the HHS fusion partner (HHS) alone were generated by recombination cloning using the Gateway cloning system. The expression vectors also encoded dihydrofolate reductase allowing selection pressure with methotrexate. Maps of the expression vectors with inserts are shown in Fig. S1, *A* and *B*. Inserts were codon-optimized for expression in hamster cells and synthesized as “DNA strings” or “gene synthesis” (Thermo Fisher Scientific). DNA string constructs were verified by DNA sequencing (Eurofins Genomics), and DNA sequences of constructs generated by gene synthesis were verified by the manufacturer.

### Suspension cell culture and protein production

Recombinant CCN2 (domains III and IV), Alb-CCN3(III), Alb-CCN5(III), and HHS proteins were produced in stably transfected ExpiCHO-S cells (Thermo Fisher Scientific) under selection pressure with methotrexate (Bio-Techne) in ExpiCHO Stable Production Medium without hypoxanthine and thymidine (Gibco, Thermo Fisher Scientific, Cat. No A3711001) supplemented with 1X GlutaMAX (Thermo Fisher Scientific, Cat. No A1286001) following generation of stably transfected cell pools.

ExpiCHO-S cells expressing the indicated proteins were seeded at 1 × 10^6^ cells/ml and fed daily with 5% EfficientFeedC + AGT Supplement 2X (Thermo Fisher Scientific, Cat. No A2503101) from day one after seeding, until harvest. Cell culture supernatants from ExpiCHO-S cells expressing CCN2, Alb-CCN3(III), and Alb-CCN5(III) proteins were harvested at day 10 to 13 when cell viabilities were >80%. ExpiCHO-S cells producing the HHS fusion partner was harvested at day 6 at a cell viability of >95%.

DG44 CHO cells (Thermo Fisher Scientific) were utilized for selection of a stable cell pool and production of HHS-CCN5(III) and HHS-CCN5(FL). For protein production, DG44 CHO cells expressing HHS-CCN5(III) or HHS-CCN5(FL) were seeded at 1 × 10^6^ cells/ml in ActiPro medium (Cytiva, Cat. No SH31037.01) supplemented with 1X GlutaMAX. After 3 days of culturing, the cells were fed daily with 4% (v/v) Cell Boost 7A (Cytiva, Cat. No SH31026.01) and 0.4% (v/v) Cell Boost 7B (Cytiva, Cat. No SH31027.01), until harvest of the cell culture supernatant at day 5. All CHO suspension cells were cultured at 37 °C at 8% CO2.

Cell culture supernatant was collected by centrifugation at 800*g* for 20 min at 4 °C before addition of PMSF (0.1 mM final concentration) and EDTA pH 8.0 (2 mM final concentration). Additionally, the cell culture supernatant from CCN2-producing cells was supplemented with MES buffer, pH 6.0 (50 mM final concentration) and L-Arginine (100 mM final concentration). The cell culture supernatants from Alb-CCN3(III)– and Alb-CCN5(III)–producing cells were supplemented with PMSF (0.1 mM final concentration) and L-arginine (100 mM final concentration). The cell culture supernatants from HHS-, HHS-CCN5(III)–, and HHS-CCN5(FL)–producing cells were supplemented with imidazole (5 mM final concentration) and ethanol to 1.5% final concentration.

### Protein purification

CCN2 protein was purified as previously described (10).

HHS-CCN5(III), HHS-CCN5(FL), and HHS protein were captured with a HisTrap Excel column (Cytiva). Peak fractions were pooled and subsequently loaded onto a size-exclusion column (Superdex 200 Increase 10/300 Gl, Cytiva).

Alb-CCN3(III) and Alb-CCN5(III) were captured using a column packed with CaptureSelect Human Albumin Affinity Matrix (Thermo Fisher Scientific) and eluted onto three HiPrep 26/10 Desalting columns connected in series (Cytiva). The eluted fractions were pooled and loaded onto a size-exclusion column (Superdex 200 Increase 26–40 or 10/300 Gl, Cytiva). Fig. S1*C* demonstrates summary of purification strategies.

Eluted fractions from the various chromatographic steps were assessed by SDS-PAGE, and the peak fractions were pooled. Protein concentrations were determined by the bicinchoninic acid (BCA) assay (Pierce BCA Protein Assay; Thermo Fisher Scientific). Following the final step of the chromatographic purification procedure of an indicated recombinant protein, purity of the protein preparation was assessed by SDS-PAGE (4–15% Mini-Criterion TGX Stain-Free Protein Gel (Bio-Rad)) and subsequent visualization and image analysis using the ChemiDoc imaging system (Bio-Rad) (Fig. S1, *D* and *E*). All chemicals and reagents used for protein purification are listed in Table S1. The final products were tested for contaminating TGF-ß1 levels, which may copurify with recombinant proteins produced in CHO cells, using DuoSet ELISA Development kit (Human TGF-ß1 DuoSet; Cat. No DY240-05 and DuoSet Ancillary Reagent Kit 1; Cat. No DY007, Bio-Techne).

### Immunoassay of phosphoproteins

Cellular contents of phospho-AKT (Ser473) in A549 cells, MCF-7 cells, or Rat2 fibroblasts following indicated treatments were determined either by a HTRF-based immunoassay (Cisbio Bioassays, Inc), by an AlphaLISA-based immunoassay (PerkinElmer), or by a Luminex bead-based immunoassay (Bioplex assay, Bio-Rad Laboratories, Inc).

For the HTRF-based immunoassay, A549 cells were seeded at a density of 10 × 10^3^ cells/well in 96-well plate coated with Fibronectin (Merck, Cat. No F1141-2 mg). Rat2 fibroblasts were seeded at a density of 2.5 × 10^3^ cells/well in 96-well plate. Following growth to approximately 80% confluency in DMEM/F12 medium with 10% FBS, the cells were serum-starved in DMEM medium without phenol red for 20 h before exposure to recombinant proteins as indicated. The cells were subsequently lysed in lysis buffer (Cisbio, Cat. No 64AKSPET and 64ERKPEG) and transferred to a HTRF 96 well-detection plate (Cisbio, Cat. No 66PL96005) for the quantitative sandwich assay of phospho-AKT (Ser473) or phospho-ERK1/2 (Thr202/Tyr204, Thr185/Tyr187), according to the manufacturer’s instructions. Readings were recorded as ratios of fluorescence emissions from the acceptor beads (665 nm) relative to the donor beads (615 nm), representing the relative energy transfer rate for each sample (accounting for varying protein contents among the samples), in a compatible HTRF plate reader (PolarStar Omega plate reader, BMG LABTECH).

For AlphaLISA-based immunoassay of phospho-AKT (Ser473) levels, A549 cells were seeded at a density of 13 × 10^3^ cells/well in 96-well plate coated with Fibronectin in DMEM/F12 medium with 10% FBS. Following growth to approximately 80% confluency, the cells were maintained in serum-free DMEM/F-12 without phenol red for 20 h before treatment for 60 min with indicated recombinant proteins. For time-course studies of CCN2-stimulated phospho-AKT and phospho-ERK1/2 levels, Rat2 fibroblasts were seeded at a density of 8 × 10^3^ cells/well and 2.5 × 10^3^ cells/well, respectively, in 96-well plates in DMEM medium with 10% FBS until 60 to 80% confluency. The cells were then maintained in DMEM with 0.1% serum and without phenol red for 20 h before treatment for indicated time points. The cells were subsequently lysed in lysis buffer provided by the AlphaLISA SureFire Ultra phospho-AKT (Ser473) and phospho-ERK1/2 (Thr202/Tyr204 and Thr185/Tyr187) Assay Kits (PerkinElmer, Cat. No ALSU-PAKT-B-HV 100 test and ALSU-PERK-A-HV 100 test), transferred to a 96-well AlphaPlate (PerkinElmer), and analyzed with the sandwich bead–based AlphaLISA immunoassay (AlphaLISA Surefire Ultra immunoassay, PerkinElmer), according to the manufacturer’s instructions. In brief, two separate antibodies towards the analyte (phosphoproteins) bring the acceptor beads in close proximity to the donor beads. Excitation of the donor beads with red light causes release of singlet oxygen which excites the acceptor beads. The intensity of fluorescence emission from the acceptor beads reflecting the amounts of analyte was recorded with a multimode plate reader (Envision Multilabel Plate Reader, PerkinElmer).

For Luminex bead-based assay of phospho-ERK1/2 (Thr202/Tyr204 and Thr185/Tyr187) and phospho-AKT (Ser473) levels in Rat2 fibroblasts, A549 cells, and MCF-7 cells following stimulations as indicated, cells were seeded at a density of 150 × 10^3^ cells/well in 6-well plates and grown to 60 to 80% confluency. The cells were subsequently starved in 0.1% FBS for 6 h before stimulation with CCN2 and indicated CCN(TSP1) fusion protein for 20 min (Fig. S2, *A* and *B*) and for 24 h (Fig. 4, *B* and *E*). The cells were subsequently lysed in BioPlex lysis buffer (Bio-Plex Pro Cell Signaling Reagent Kit, Bio-Rad Cat. No 171304006M), and the samples were corrected for protein content (Pierce BCA assay kit Cat. No 23225, Thermo Fisher Scientific) and subjected to assay of phospho-ERK1/2 and phospho-AKT contents according to the manufacturer's instructions.

### Real-time RT-PCR

MDA-MB-231 cells were seeded at a density of 150 × 10^3^ cells/well in 12-well plates, propagated to approximately 80% confluency, and subsequently serum-starved in 0.1% FBS overnight. Then, following 48 h of stimulation with indicated protein, total RNA was extracted from the cells by the RNeasy mini-kit (QIAGEN) according to the manufacturer’s instructions. One microgram of total RNA from each sample was reverse-transcribed with the TaqMan Reverse Transcription Reagents Kit (Thermo Fisher Scientific, Cat. No N8080234), according to the manufacturer’s protocol, and the cDNA generated was utilized for real-time qPCR analyses using TaqMan Gene Expression Assays, Fast Advanced Master Mix, and the QuantStudio 12K Flex Real-Time PCR cycler (Thermo Fisher Scientific). The mRNA levels indicated show the abundance of the target genes relative to that of the housekeeping control (GAPDH) mRNA. The TaqMan probes used were as follows: Human ESR1/ER-α (Assay ID: Hs01046816_m1), Human CDH1/E-cadherin (Assay ID: Hs01023895_m1), Human ID2 (Assay ID: Hs04187239_m1), and Human GAPDH (Assay ID: Hs02786624_g1).

### *In vitro* cell scratch assay

Rat2 fibroblasts or primary human lung fibroblasts were seeded at a density of 100 × 10^3^ cells/well in 12-well cell culture plates in DMEM containing 10% FBS. When the cells had grown to near confluency, the cells were further maintained for 16 to 20 h in serum-free DMEM. The cells were subsequently washed in PBS, and the cell layer was subsequently artificially injured by scratching the plate with a 1000 μl tip (Rat2 fibroblasts) and 10 μl tip (primary human lung fibroblasts) with the tip perpendicular to plate surface. The PBS was replaced with serum-free DMEM medium, and stimulants (recombinant proteins) as indicated were added. The cells were incubated another 24 h before termination of the experiment. The cells were then washed carefully in PBS, fixed with 4% paraformaldehyde, and permeabilized with 0.1% Triton-X100. The cells were subsequently stained with Hoechst 33258 (Hoechst 33258, pentahydrate (bis-Benzimide), Thermo Fisher Scientific) for 15 min and washed properly in PBS for visualization of nuclei. Images were taken by a Zeiss Axio Observer Inverted Microscope at a 5× magnification power. The gap between the leading edges of the scratch wound was measured with a ruler in each 5× field and three measures recorded from each image using ZEN 3.1 (ZEN lite, blue edition) software (https://www.zeiss.com/microscopy). The average of measurements in μm for each well was used for data analysis. Photomicrographs in figures are shown in grayscale using Adobe Photoshop CS3.

### Cell migration assays

Transwell migration assays (modified Boyden chamber assays) were performed in two different formats. The assays were either performed with Millicell Cell Culture Inserts (Merck Life Science) for 24-well cell culture plates or as a multiscreen-based migration assay (96-well Multiscreen-MIC plate). For the former migration assays (24-well microporous cell culture inserts), Rat2 fibroblasts or primary human lung fibroblasts were seeded in serum-free DMEM or Fibroblast Growth Basal medium at a cell density of 1 × 10^5^ and 3 × 10^4^ respectively, in 3 μm- (Rat2 fibroblasts) or 5 μm- (primary human lung fibroblasts) pore-diameter Millicell Cell Culture Inserts (Cat. No PITP01250 and Cat. No CLS3421, Merck Life Science). The inserts were placed in transwell dishes, and stimulants were added to the lower chamber of the transwells. Following 20 h incubation, the cells were fixed with 4% paraformaldehyde and permeabilized with 0.1% Triton-X100. Cells that had not migrated from the top chamber were gently scraped off from the top side of the membrane using cotton swabs. Cells that had migrated through the porous membrane and were residing on the bottom side of the porous membrane were stained by soaking inserts in Hoechst 33258 for 15 min before washing with PBS. Afterward, the membranes were cut out from inserts and immobilized on glass slides using one drop of ProLong Gold Antifade Mountant (Thermo Fisher Scientific) and cover slips. Images were taken at 10× magnification power by a Zeiss Axio Observer Inverted Microscope. Migrated cells were counted using ImageJ 1.51K (NIH) software (https://imagej.nih.gov), and 6-12 10× fields from each membrane were counted to determine the average cell number migrated in each experiment. Total of 72 10× fields were counted per condition. Images were then converted to grayscale (black and white) mode using Adobe Photoshop CS3.

For multiscreen-based migration assays, Rat2 fibroblasts were starved in 0.1% FBS for 6 h, thereafter washed with PBS and seeded in serum-free DMEM medium at a cell density of 25 × 10^3^/well on top of 5 μm pore-diameter Multiscreen-Mic 96 well-plates (Merck, Cat. No MAMIC5S10). Stimulants in serum-free medium were added to the lower chamber of the Multiscreen-MIC plates. Following 20 h of incubation, cells that had migrated through the porous membrane and were residing on the lower side of the membranes or in the lower trays were detached using Accutase (VWR, Cat. No L0950-100) at 37 °C for 30 min with gentle shaking, collected, and transferred to wells of black-walled plates. Quantitative analysis of migrated cells was performed with CellTiter-Glo 2.0 Cell Viability Assay (Promega, Cat. No G9242). Luminescent signals were recorded using the PolarStar Omega plate reader (BMG LABTECH).

### Western blot analysis

Rat2 fibroblasts were seeded in 6-well cell culture plates at a density of 150 × 10^3^ cells/well in DMEM containing 10% FBS and maintained overnight until 60% confluency. The cells were subsequently serum-starved in DMEM containing 0.1% FBS for 6 h before exposure to indicated recombinant proteins for 24 h in serum-free DMEM. The cells were subsequently lysed in hypotonic buffer containing 10 mM Tris–HCl, pH 8.8, 1% SDS, 2 mM sodium orthovanadate (Na_3_VO_4_), 10 mM sodium fluoride (NaF), 4 mM β-glycerophosphate, and 4 mM pyrophosphate (all chemicals analytical grade from Merck), sonicated, and centrifuged at 14000*g* for 30 min. Protein concentrations of the samples were determined with the Pierce BCA assay kit (Thermo Fisher Scientific, Cat. No 23225). Equal amounts of protein from each sample were separated by SDS-PAGE (4–15% TGX gradient gels, Bio-Rad Laboratories) followed by transfer to PVDF membranes using the Trans-Blot Turbo semidry blotting system (Bio-Rad). Membranes were blocked in 5% nonfat dry milk dissolved in Tris-buffered saline with Tween 20 (20 mM Tris–HCl, pH 7.4, 140 mM NaCl, 2.5 mM KCl, and 0.1% Tween 20) for 1 h before probing with primary antibody overnight at 4 °C. Membranes were incubated with primary antibody against phospho-ERK1/2 (Thr202/Tyr204 and Thr185/Tyr187) (Cell Signaling, Cat. No 9101, USA) or total ERK1/2 (Cell Signaling, Cat. No 9102). Secondary antibody incubation was performed at room temperature for 1 h with HRP-linked anti-rabbit IgG (Cell Signaling, Cat. No 7074). Immunoreactivity was visualized with Clarity Western ECL Substrate (Bio-Rad, Cat. No 1705060) and recording of chemiluminescence using the ChemiDoc imaging system (Bio-Rad). Densitometric analyses of immunoreactive bands were performed using the Image Lab 6.0.1 software (https://www.bio-rad.com).

### Collagen gel contraction assay

A suspension of Rat2 fibroblasts in DMEM (lacking phenol red) containing 0.5% FBS to be seeded at a density of 1.5 × 10^5^ cells/well were prepared in 0.4 ml in an Eppendorf tube and were mixed well with 0.2 ml pH-adjusted collagen solution (3 mg/ml collagen I, rat tail; Thermo Fisher Scientific, Cat. No A1048301). The mixture was immediately added to a 24-well culture plate and allowed to solidify at 37 °C incubator for 45 min. After the collagen gel had formed, it was released gently by running a 200 μl pipet tip along the peripheral gel edges and thereafter allowed to float in DMEM 1× containing 0.5%-serum. Following addition of stimulants, the plate was incubated for 4 days. Then, MTT (thiazolyl blue tetrazolium bromide; 3-[4, 5-dimethylthiazol-2-yl]-2, 5-diphenyltetrazolium bromide; Merck Life Sciences) solution at a final concentration of 0.25 mg/ml was added to the wells before imaging of the gels by the Oxford Optronix Gelcount system. The ImageJ software was used to calculate the area of collagen gels and to analyze gel contraction. Images were then adjusted for brightness/contrast using Adobe Photoshop CS3.

### Mammosphere assay

In order to make single-cell suspensions of MCF-7 human mammary adenocarcinoma cells, the cells were trypsinized followed by aspiration through a 25-gauge needle for three times and filtering through a 40-μm membranous cell filter before seeding 200 cells/well in 96-well ultralow-binding plates (Sarstedt, Cat. No 83.3924.500) to allow adhesion-independent growth in DMEM/F-12 supplemented with B-27 without vitamin A (Thermo Fisher Scientific; Cat. No 17504044). Following addition of stimulants to the cells, the plate was incubated for 7 days and subsequently stained with MTT (thiazolyl blue tetrazolium bromide) solution. The number of spheres formed was semiautomatically quantified by the Oxford Optronix Gelcount system.

### Statistical analysis

Data are shown as the mean ± SD or mean ± SEM as indicated in the legends to the figures. Statistical analyses were performed using GraphPad Prism, version 6.0. Statistical analysis of differences between two groups was assessed by unpaired Student’s two tailed *t* test. Statistical analysis of differences among several experimental groups was performed by one-way ANOVA, followed by Dunnett’s or Šidák’s post hoc test. Statistical analysis with the Holm- Šidák method was used as indicated in the figure legends. *p* < 0.05 was considered to indicate statistically significant differences.

## Data availability

The source data of all figures and presentations in this report are available from the corresponding author upon request.

## Supporting information

This article contains supporting information.

## Conflict of interest

Ole Jørgen Kaasbøll and Håvard Attramadal are named inventors in a patent covering the commercial use of fusion proteins containing the TSP1-repeat homology domain of CCN proteins and are both shareholders of Tribune Therapeutics AB, the company that has licensed the patent. All other authors declare that they have no conflicts of interest with the contents of the article.
