# Supplementary material for: The carboxyl-terminal TSP1-homology domain is the biologically active effector peptide of matricellular protein CCN5 that counteracts profibrotic CCN2
Source: J Biol Chem. 2022 Dec 15;299(1):102803. doi: 10.1016/j.jbc.2022.102803 (PMC9860493; doi:10.1016/j.jbc.2022.102803)
Supplement: Supplemental Figures S1–S4 and Table S1 [file mmc1.pdf]

The carboxyl-terminal TSP1-homology domain is the biologically active effector peptide of matricellular protein CCN5 that counteracts profibrotic CCN2

Sima Zolfaghari[1, 2], Ole Jørgen Kaasbøll[1#], Vivi T. Monsen[1, 2], Bojana Sredic[1], Else Marie V. Hagelin[1#], and Håvard Attramadal[1, 2]

[1)] Institute for Surgical Research, Oslo University Hospital, Oslo, Norway [2)] Institute of Clinical Medicine, University of Oslo, Norway

[#]**Footnote - Present address:** Tribune Therapeutics AS, c/o Sharelab, Gaustadalléen 21, 0349 OSLO, Norway.

Supporting information

Contains supplementary Figures S1, S2, S3, S4 and Table S1 with figure legends.

Supplementary Figures and Tables

Fig. S1

A Destination vector

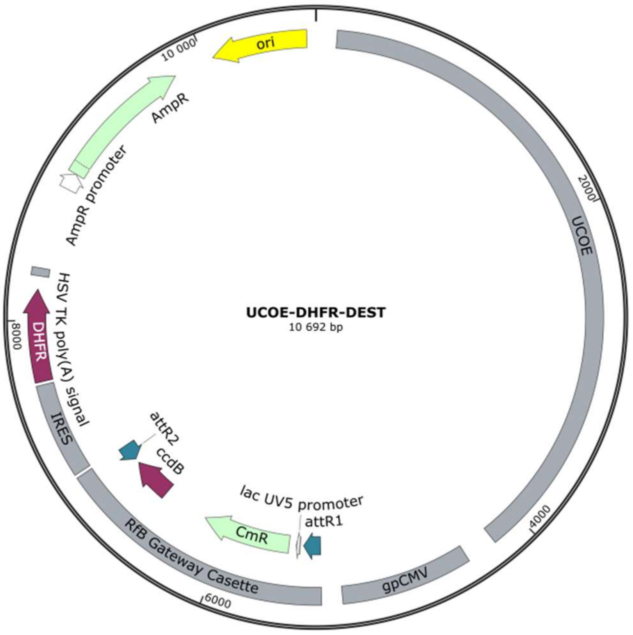

Protein inserts

B-1 HHS-CCN5(III)

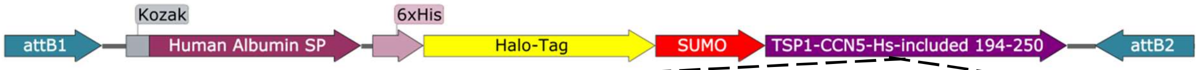

CPEWSTAWGPCSTTCGLGMATRVSNQNRFCRLETQRRLCLSRPCPPSRGRSPQNSAF

B-2 HHS-CCN5(FL)

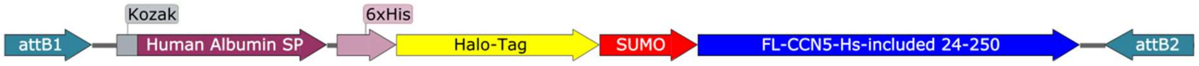

B-3 Alb-CCN5(III)

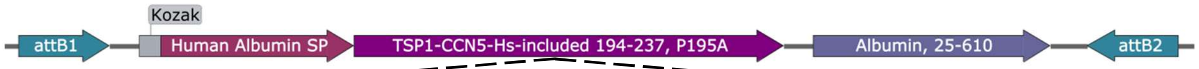

CAEWSTAWGPCSTTCGLGMATRVSNQNRFCRLETQRRLCLSRPC

B-4 Alb-CCN3(III)

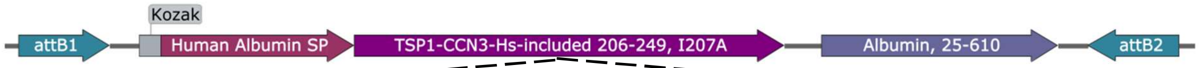

CAEQTTEWTACSKSCGMGFSTRVTNRNRQCEMLKQTRLCMVRPC

C Purification scheme

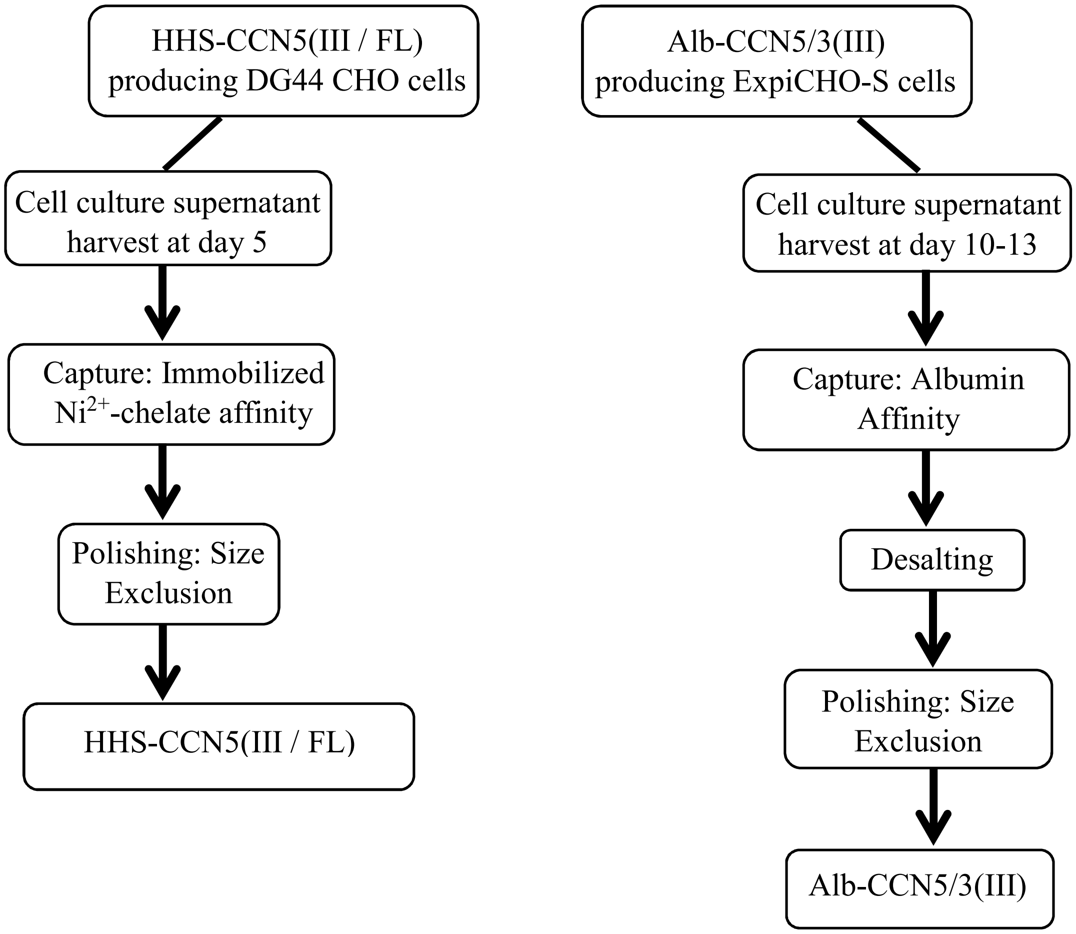

SDS-PAGE of recombinant proteins

D **E**

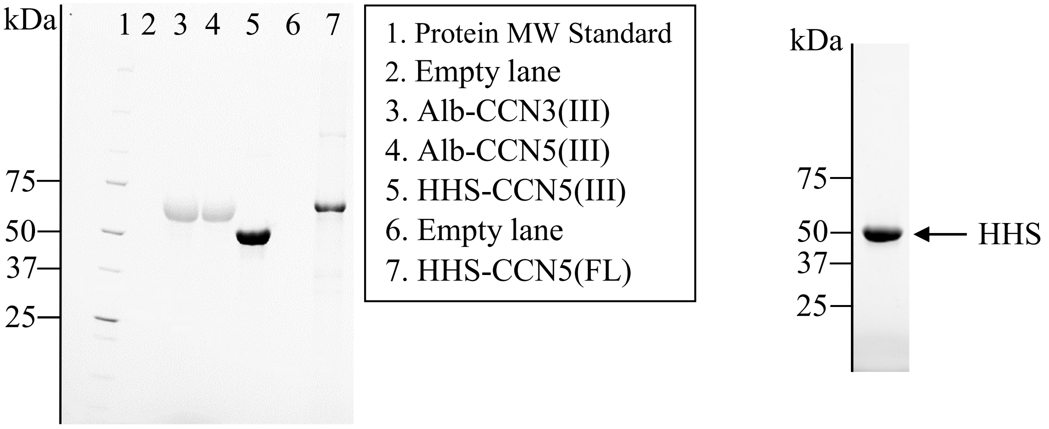

Legend to Supplementary Fig. S1. Schematics of expression vector constructs for generation of stably transfected cell clones, schemes for chromatographic purification of recombinant proteins, and verification of purified recombinant proteins.

Panel (A). Schematic illustrating the UCOE-DHFR-DEST destination vector used for generation of all expression vectors constructs for stable expression of recombinant proteins in ExpiCHO-S and DG44 CHO cells in this study. All open reading frames encoding recombinant fusion proteins have been constructed with Kozak consensus sequence for initiation of translation at the 5'-end and an IRES-element (Internal Ribosomal Entry Site) and cDNA encoding dihydrofolate reductase (DHFR) inserted at the 3'-end for selection and amplification of stable cell clones with methotrexate. Panels (B1-B4). Schematics illustrating the DNA constructs and encoded CCN peptide sequences for His-Halo-Sumo-CCN5(III) (B-1), His-Halo-Sumo-CCN5(FL) (B-2), Albumin-CCN5(III) (B-3) and Albumin-CCN3(III) (B-4). All DNA constructs were flanked by Gateway recombination sequences for directional, ligation-independent cloning into the destination expression vector. The amino acid numbering of the human CCN5 and CCN3 fragments are according to Uniprot IDs O76076 (CCN5; B-1, B-2, B-3), and P48745-1 (CCN3; B4), respectively. The signal peptide (SP) for secretion including cleavage site from human albumin was used for all the constructs and appended N-terminally as indicated. All the schematic maps were created with the SnapGene program version 5.3.2. Panel (C). Schematic of the purification scheme of the various recombinant CCN fusion proteins. Panels (D and E). Verification of purified recombinant fusion proteins (D), and the HHS fusion partner (E), and assessment of purity following separation of the purified proteins by SDS-polyacrylamide gel electrophoresis using 4–15% gradient Mini-Criterion TGX Stain-Free Protein Gels (Bio-Rad). The protein MW standards used in Panel (D) were the Precision Plus Protein Unstained Standards (Bio-Rad). Panel (E) shows cropped lane from SDS-polyacrylamide gel electrophoresis of HHS fusion partner with MW marker values indicated.

Fig. S2

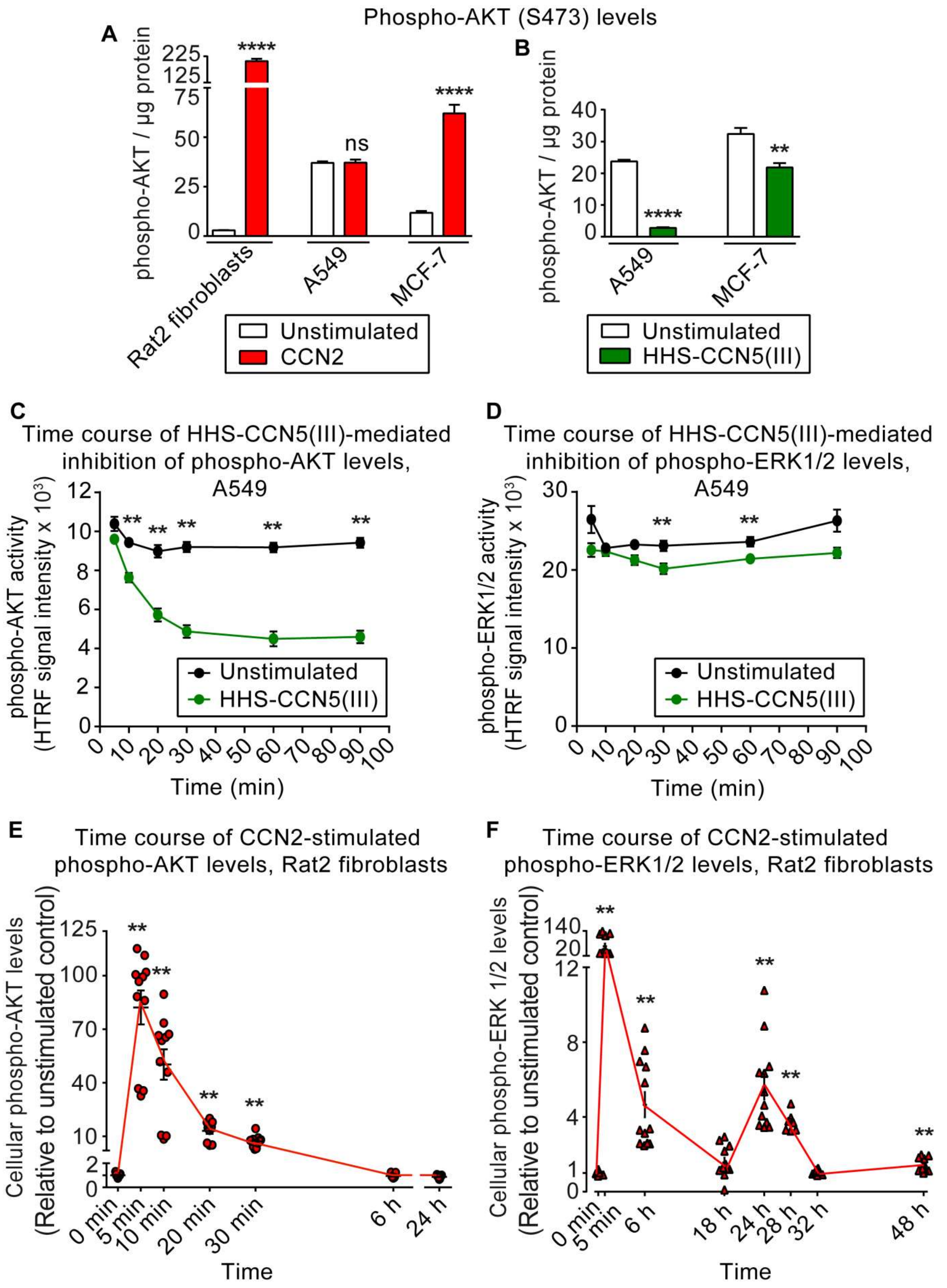
Phospho-AKT (S473) levels
A
phospho-AKT / µg protein
225
125
75
50
25
0
****
ns
****
Rat2 fibroblasts
A549
MCF-7
Unstimulated
CCN2
B
phospho-AKT / µg protein
40
30
20
10
0
****
**
A549
MCF-7
Unstimulated
HHS-CCN5(III)
C
Time course of HHS-CCN5(III)-mediated inhibition of phospho-AKT levels, A549
phospho-AKT activity (HTRF signal intensity x 10³)
Unstimulated
HHS-CCN5(III)
Time (min)
D
Time course of HHS-CCN5(III)-mediated inhibition of phospho-ERK1/2 levels, A549
phospho-ERK1/2 activity (HTRF signal intensity x 10³)
Unstimulated
HHS-CCN5(III)
Time (min)
E
Time course of CCN2-stimulated phospho-AKT levels, Rat2 fibroblasts
Cellular phospho-AKT levels (Relative to unstimulated control)
0 min
5 min
10 min
20 min
30 min
6 h
24 h
Time
F
Time course of CCN2-stimulated phospho-ERK1/2 levels, Rat2 fibroblasts
Cellular phospho-ERK 1/2 levels (Relative to unstimulated control)
0 min
5 min
6 h
18 h
24 h
28 h
32 h
48 h
Time

Legend to Supplementary Fig. S2. CCN2-stimulated phospho-AKT and phospho-ERK 1/2 activities in Rat2 fibroblasts, A549, and MCF-7 cells and inhibition by HHS-CCN5(III).

Panel (A) demonstrates histogram of phospho-AKT (Ser473) activities of Rat2 fibroblasts, A549, and MCF-7 cells stimulated with CCN2 (5 µg/ml) for 20 min. Cellular contents of phospho-AKT (Ser473) were assayed with a Luminex-based immunoassay (Bio-Plex; Bio-Rad). The data represent the mean ± SEM ($n \geq 3$ independent experiments assayed in duplicate for each condition). Statistical analysis was performed with one-way ANOVA and Sidak's post hoc test. ****$P < 0.0001$; ns: indicates not statistically significant vs. control group.

Panel (B) demonstrates histogram of phospho-AKT (Ser473) activities of A549, and MCF-7 cells following exposure to HHS-CCN5(III) (100 µg/ml) for 20 min. Cellular contents of phospho-AKT (Ser473) were assayed with a Luminex-based immunoassay (Bio-Plex; Bio-Rad). The data represent the mean ± SEM ($n \geq 3$ independent experiments assayed in duplicate for each condition). Statistical significance was assessed by one-way ANOVA and Sidak's post hoc test. **$P < 0.01$; ****$P < 0.0001$ vs. control group.

Panels (C and D) demonstrate time course of HHS-CCN5(III) (100 µg/ml)-mediated inhibition of phospho-AKT (Ser473) and phospho-ERK1/2 (Thr202/Tyr204 and Thr185/Tyr187) levels in A549 cells. The panels demonstrate time-dependent inhibition of cellular phospho-AKT (Ser473) (panel (C)), and phospho-ERK1/2 (Thr202/Tyr204 and Thr185/Tyr187) (panel (D)) levels. The data represent the mean ± SEM ($n = 3$ independent experiments assayed in 4 replicates for each condition). Statistical analysis were determined using the Holm-Sidak method with **$P < 0.01$ significance level vs. control group.

Panels (E and F) demonstrate scatter plots of time course of phospho-AKT (Ser473) and phospho-ERK1/2 (Thr202/Tyr204 and Thr185/Tyr187) levels in Rat2 fibroblasts following stimulation with CCN2 (5 µg/ml). Panel (E) shows rapid increase of phospho-AKT activities following stimulation with CCN2 with peak levels at about 5 min and subsequently decreasing swiftly towards basal levels. The data represent the mean ± SD ($n = 3$ independent experiments assayed in 3-4 replicates for each condition). Statistical analysis was performed using the Holm-Sidak method with statistical significance representing **$P < 0.01$ vs. control group. Panel (F) shows rapid increase of phospho-ERK1/2 activities following stimulation with CCN2 (5 µg/ml) with peak levels at about 5 min. Activities returned rapidly towards basal levels, but a secondary peak of activities was observed around 24 hrs of stimulation. The data represent the mean ± SD ($n = 3$ independent experiments assayed in 2-4 replicates for each condition). Statistical analysis

were determined using the Holm-Sidak method with **$P < 0.01$ significance level vs. control group.

Fig. S3

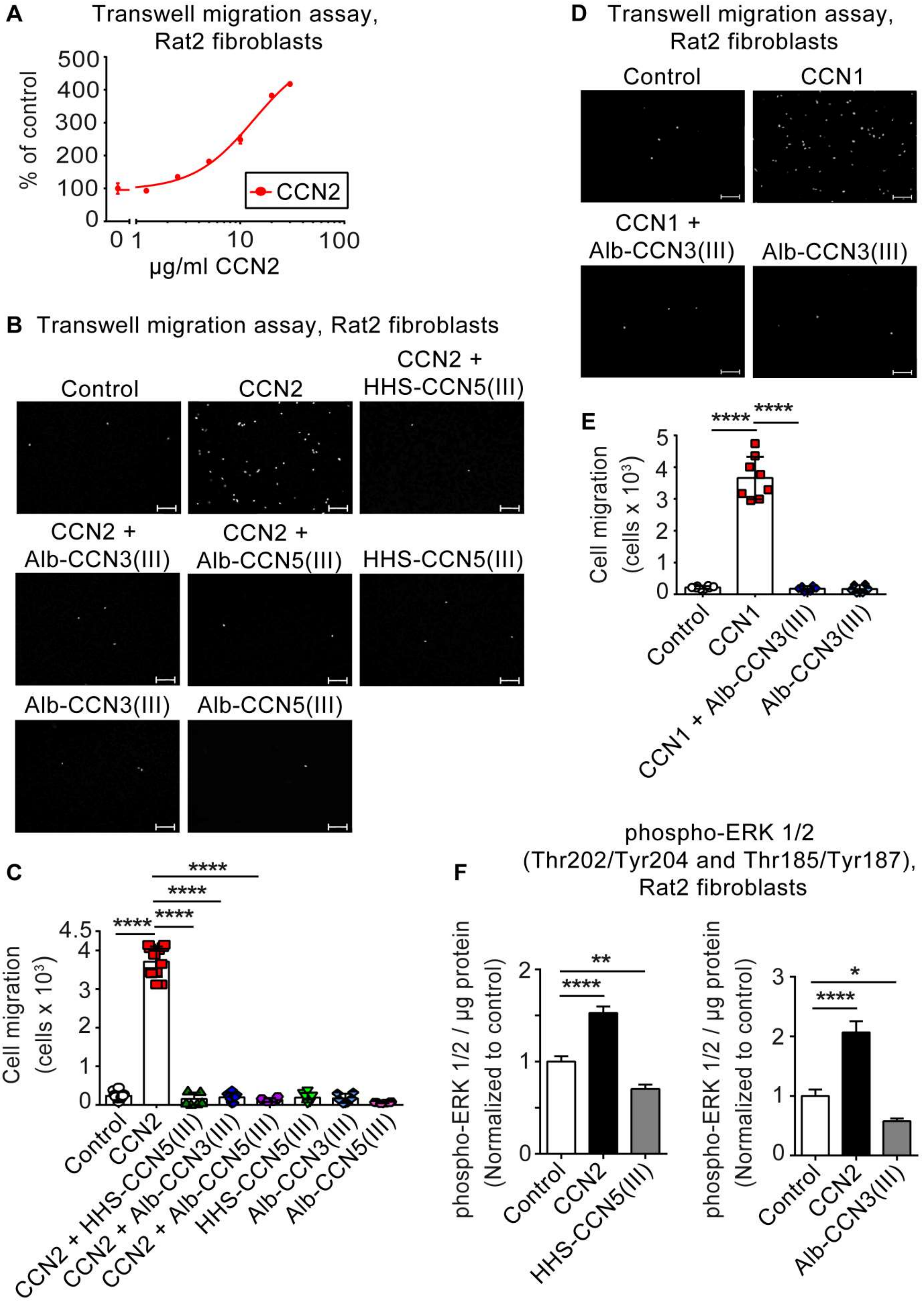
A
Transwell migration assay,
Rat2 fibroblasts
% of control
500
400
300
200
100
0
0 1
10
100
μg/ml CCN2
CCN2
B Transwell migration assay, Rat2 fibroblasts
Control
CCN2
CCN2 +
HHS-CCN5(III)
CCN2 +
Alb-CCN3(III)
CCN2 +
Alb-CCN5(III)
HHS-CCN5(III)
Alb-CCN3(III)
Alb-CCN5(III)
C
Cell migration
(cells x 10³)
4.5
4
3
2
1
0
****
Control
CCN2
CCN2 + HHS-CCN5(III)
CCN2 + Alb-CCN3(III)
CCN2 + Alb-CCN5(III)
HHS-CCN5(III)
Alb-CCN3(III)
Alb-CCN5(III)
D Transwell migration assay,
Rat2 fibroblasts
Control
CCN1
CCN1 +
Alb-CCN3(III)
Alb-CCN3(III)
E
Cell migration
(cells x 10³)
5
4
3
2
1
0
****
Control
CCN1
CCN1 + Alb-CCN3(III)
Alb-CCN3(III)
F
phospho-ERK 1/2
(Thr202/Tyr204 and Thr185/Tyr187),
Rat2 fibroblasts
phospho-ERK 1/2 / μg protein
(Normalized to control)
2
1
0
**
****
Control
CCN2
HHS-CCN5(III)
phospho-ERK 1/2 / μg protein
(Normalized to control)
3
2
1
0
*
****
Control
CCN2
Alb-CCN3(III)

Legend to Supplementary Fig. S3. Concentration-effect curve of CCN2-stimulated cell migration, CCN3(III)- or CCN5(III)-mediated inhibition of CCN2 or CCN1 cell migration, and phospho-ERK 1/2 activities in Rat2 fibroblasts.

Panel (A) shows concentration-effect curve of CCN2-stimulated cell migration of Rat2 fibroblasts following 20 h incubation in the presence of increasing concentrations of CCN2 using a multiscreen transwell migration assay (modified Boyden chamber). The figure shows CCN2-stimulated cell migration as percent of cell migration of unstimulated cells. Data points represent the mean ± SEM (n = 3 independent experiments assayed in three replicates for each condition). The data were subjected to curve fitting by four-variable nonlinear regression in GraphPad Prism. EC_{50} for CCN2-stimulated migration of Rat2 fibroblasts is approx. 13 µg/ml.

Panel (B) shows representative photomicrographs of transwell migration assay (Boyden chamber principle) of Rat2 fibroblasts stimulated in the absence or presence of CCN2 (5 µg/ml) and HHS-CCN5(III) (100 µg/ml), Alb-CCN3(III) (60 µg/ml), or Alb-CCN5(III) (60 µg/ml) for 20 h. The cells that had migrated through the semi-permeable membrane were stained with Hoechst dye 33258 and counted. Scale bar is 100 µm. Panel (C) shows scatter-plot of quantitative analysis of transwell cell migration assay. The data represent the mean ± SD (n ≥ 3 independent experiments assayed in duplicate for each condition). The data were subjected to statistical analysis by one-way ANOVA with Šidák's post hoc test. ****p < 0.0001 vs. CCN2 group.

Panel (D) demonstrates representative photomicrographs of transwell migration assay (Boyden chamber principle) of Rat2 fibroblasts stimulated in the absence or presence of CCN1 (5 µg/ml) with or without co-incubation with Alb-CCN3(III) (60 µg/ml) for 20 h. The cells that had migrated through the semi-permeable membrane were stained with Hoechst dye 33258 and counted. Scale bar is 100 µm. Panel (E) shows scatter-plot of quantitative analysis of transwell cell migration. The data represent the mean ± SD (n ≥ 3 independent experiments assayed in duplicate for each condition). The data were subjected to statistical analysis by one-way ANOVA with Šidák's post hoc test. ****p < 0.0001 vs. CCN1 group.

Panel (F) demonstrates histogram of Luminex immunoassay (BioPlex) of phospho-ERK1/2 (Thr202/Tyr204 and Thr185/Tyr187) activities of non-confluent Rat2 fibroblasts stimulated in the absence or presence of CCN2 (5 µg/ml), HHS-CCN5(III) (50 µg/ml) or Alb-CCN3(III) (60 µg/ml) for 24 h. The data represent the mean ± SEM (n = 3 independent experiments assayed in triplicate for each condition). The data were subjected to statistical analysis by one-way

ANOVA with Dunnett's post hoc test. *P < 0.05; **P < 0.01; ****P < 0.0001 vs. control group. The figure shows that CCN2 stimulates cellular phospho-ERK1/2 contents, while both HHS-CCN5(III) and Alb-CCN3(III) reduce basal (unstimulated) levels of phospho-ERK1/2.

Fig. S4

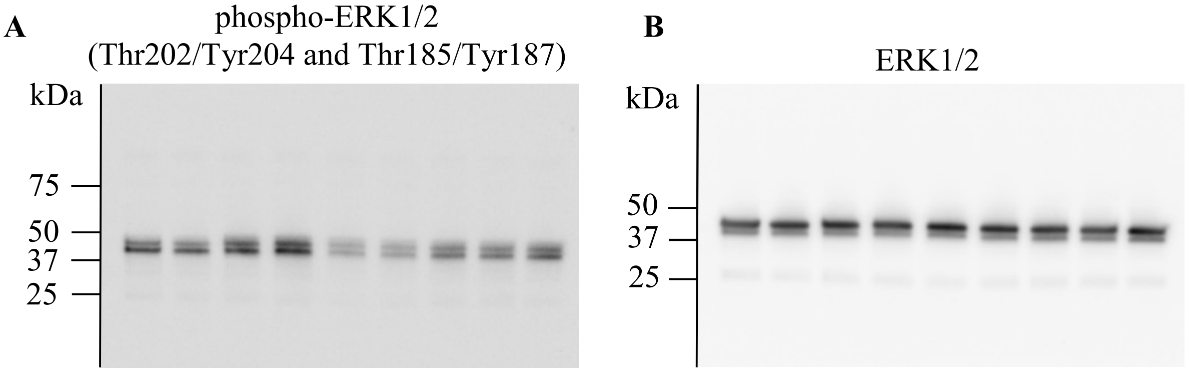

Legend to Supplementary Fig. S4. Uncropped blots.

Panels (A and B) demonstrate uncropped blots from Fig. 3C with molecular weight marker values indicated.

Table. S1

Chromatographic column matrices

Protein	Chromatography step	Column	Binding buffer	Elution buffer
HHS, HHS-CCN5(III) and HHS-CCN5(FL)	Immobilized Ni^{2+} Affinity	HisTrap Excel	20 mM Hepes pH 7.5, 50mM NaCl, 5mM Imidazole	20 mM Hepes pH 7.5, 50mM NaCl, 250mM Imidazole
	Size Exclusion	Superdex 200 Increase 10/300 GL	N/A	20 mM Hepes pH 7.5, 50mM NaCl
Alb-CCN5(III) and Alb-CCN3(III)	Albumin Affinity	CaptureSelect Human Albumin Affinity Matrix	100mM $Na_{1/2}H_{1/2}PO_4$, 100mM L-Arginine, pH 6.50	30 mM Citratic acid, 500mM L-Arginine, pH 3.50
	Desalting and Size Exclusion	HiPrep 26/10 Desalting, Superdex 200 Increase 26-40 or Superdex 200 Increase 10/300 GL	N/A	100mM $Na_{1/2}H_{1/2}PO_4$, 100mM L-Arginine, pH 6.50

Legend to Supplementary Table S1. Details of the chromatographic column matrices and buffers used in purification of the recombinant proteins.
